# Investigating the pathogenic role of calpain proteases and the therapeutic potential of their inhibition in mice modelling Machado-Joseph disease

**DOI:** 10.1093/hmg/ddaf196

**Published:** 2026-01-06

**Authors:** Katherine J Robinson, Amanda L Wright, Maxinne Watchon, Andrea Kuriakose, Ignacio Simo, Angela S Laird

**Affiliations:** Motor Neuron Disease Research Centre, Macquarie Medical School, Macquarie University, Level 1, 75 Talavera Road, Sydney, NSW 2109, Australia; Motor Neuron Disease Research Centre, Macquarie Medical School, Macquarie University, Level 1, 75 Talavera Road, Sydney, NSW 2109, Australia; School of Rural Medicine, Charles Sturt University, Level 1, 1521 Forest Road, Orange, NSW 2800, Australia; Motor Neuron Disease Research Centre, Macquarie Medical School, Macquarie University, Level 1, 75 Talavera Road, Sydney, NSW 2109, Australia; Motor Neuron Disease Research Centre, Macquarie Medical School, Macquarie University, Level 1, 75 Talavera Road, Sydney, NSW 2109, Australia; Motor Neuron Disease Research Centre, Macquarie Medical School, Macquarie University, Level 1, 75 Talavera Road, Sydney, NSW 2109, Australia; Motor Neuron Disease Research Centre, Macquarie Medical School, Macquarie University, Level 1, 75 Talavera Road, Sydney, NSW 2109, Australia

**Keywords:** Calpains, Neurodegeneration, Preclinical Drug Testing, Calpain Inhibitor Compounds

## Abstract

Machado-Joseph disease (MJD, also known as spinocerebellar ataxia type-3) is a fatal disease characterised by motor impairments and the presence of aggregated ataxin-3, the protein affected in MJD, in degenerating brain regions. Ataxin-3 protein aggregates have previously been reported to contain both full-length ataxin-3 protein and shorter protein fragments, highlighting proteolytic cleavage as a pathogenic mechanism. Calpains, calcium-activated proteases, have been reported to cleave ataxin-3 and have been implicated in MJD pathogenesis. This study aimed to explore whether calpain proteases were overactive at early, pathogenesis-relevant timepoints in male transgenic CMVMJD135 mice modelling MJD and identify the timepoint of calpain overactivation through obtaining longitudinal plasma samples. We detected increased levels of cleaved αII-spectrin in plasma from MJD mice as early as 12 weeks of age, shortly after the onset of neurological symptoms. Cerebellar and brainstem tissue from 15-week-old mice was immunoblotted, revealing a trend towards increased levels of calpain 1, and increased cleavage of calpain substrates such as αII-spectrin, beclin-1 and TAR DNA binding protein 43 (TDP-43) within the cerebellum. Further, we found that short-term treatment of male MJD mice (from 10 to 12 weeks of age) with the calpain inhibitor compound calpeptin yielded improvements in neurological symptoms and reduced the presence of cleaved αII-spectrin in plasma and cerebellum tissue when compared to vehicle treated MJD males. Our findings suggest that calpain overactivity may be an early disease phenotype that contributes to neurodegeneration in transgenic CMVMJD135 mice modelling MJD, and that calpeptin warrants further investigation as a potential treatment for MJD.

## Introduction

Machado-Joseph disease (MJD, also known as spinocerebellar ataxia type-3) is one of the most prevalent forms of hereditary ataxia found globally [1]. MJD is characterised by motor impairments, dystonia, ataxic gait, paralysis leading to wheelchair dependence and other neurological perturbations such as visual disturbances, insomnia, nystagmus and cognitive changes [2–7]. MJD is caused by autosomal dominant inheritance of an abnormal expansion of the trinucleotide (CAG) repeat region of the *ATXN3* gene found on chromosome 14 [8, 9]. In healthy individuals, the CAG repeat region is typically between 12 and 44 repeats, whilst inheritance of *ATXN3* with 54 or more repeats can lead to the development of neurological deficits [10–12]. Sadly, there is a current lack of treatments to slow or prevent the development of MJD, with most patients succumbing to the disease.

There is a strong correlation between the length of the inherited mutation and disease severity and mortality age [10–13]. The *ATXN3* gene encodes the protein ataxin-3, with the CAG repeat region encoding a long string of glutamine amino acids, known as the polyglutamine region [10]. Mutant expansion of the polyglutamine region has been shown to alter the function of ataxin-3 [14] and increase the propensity for aggregation of the ataxin-3 protein [15–17]. Indeed, ataxin-3 polyglutamine expansions cause significant degeneration throughout the central nervous system (CNS), with neuronal loss and the presence of ataxin-3 positive protein inclusions within neuronal nuclei in regions such as the brainstem [18], cerebellum [19, 20] and spinal cord [21].

Proteolytic cleavage by proteases, enzymes that act to cleave full-length proteins into smaller protein fragments [22], has been identified as a mechanism that may contribute to protein aggregate formation [23]. Proteases play an important physiological role in regulating protein activity and cellular functions; however, aberrant activity of proteases may contribute to proteotoxicity and neurodegenerative processes [23–26]. One protease family that has been implicated in the pathogenesis of many neurodegenerative diseases are the calpains, calcium-activated proteases. The most highly expressed calpain isoforms within the CNS are calpain 1 (*CAPN1*) and calpain 2 (*CAPN2*), which are activated by elevations in intracellular calcium signalling [26, 27]. The activity of calpains is tightly regulated through calpastatin (*CAST*), the only known endogenous inhibitor of calpain activity [28]. Calpains are known to cleave many essential proteins including cytoskeletal proteins such as α-spectrin/α-fodrin [29] and tau [30], autophagy related proteins such as beclin-1/ATG6 [31] and p62/SQSTM1 [32], and apoptosis related proteins caspase 3 and PARP1 [33]. Overactivity of calpains, and subsequent aberrant cleavage of disease-associated proteins, has been implicated in many neurodegenerative diseases including Parkinson’s disease [34, 35], Alzheimer’s disease [30, 36], motor neuron disease [37], Huntington’s disease [38] and spinocerebellar ataxia type-17 [39]. These findings highlight that calpain overactivity may be a disease mechanism common to many neurodegenerative diseases, irrespective of any underlying genetic cause.

Proteolytic cleavage of ataxin-3 was first reported by Goti *et al* [40] who detected the presence of both full-length ataxin-3 and shorter ataxin-3 fragments in ataxin-3 positive protein aggregates from MJD patient brain tissue. There is experimental evidence to suggest that calpains may be the key protease responsible for fragmentation or cleavage of ataxin-3, including calpain-mediated cleavage in cultured cells expressing mutant ataxin-3 [41], MJD patient induced pluripotent stem cells (IPSCs) [42] and transgenic zebrafish expressing mutant human ataxin-3 with 84 glutamines [43]. The precise site of calpain cleavage within wildtype ataxin-3 and mutant expanded ataxin-3 has also been investigated [44]; however, the precise mechanism triggering calpain overactivity remains unclear.

It has been hypothesised that alterations to calpastatin could contribute to the dysregulated activity of calpains in MJD. Examination of calpastatin levels in post-mortem brain tissue from MJD patients have revealed downregulation of calpastatin expression in some affected brain regions; however, the degree of downregulation varies across patients [45]. Further, knockout of calpastatin decreased lifespan and increased the presence of ataxin-3 aggregates in transgenic mice expressing ataxin-3 with 140Q [24], whilst overexpression of calpastatin in another MJD mouse model decreased the number and size of ataxin-3 inclusions and prevented neuronal loss [45].

This study aims to identify whether calpain proteases are overactive in male CMVMJD135 mice modelling MJD at early disease stages, indicating potential implications in disease pathogenesis and therefore warranting investigation for therapeutic modulation. We chose to examine this phenotype exclusively in male mice as previous work from our laboratory has shown male CMVMJD135 mice display a more severe disease progression [46]. We aimed to identify the timepoint at which the calpain system becomes dysregulated by collecting repeated blood samples from the same cohort of mice and examining plasmatic levels of cleaved αII-spectrin. Cerebellar and brainstem tissue was harvested after euthanasia and used for immunoblotting of calpain 1, calpain 2, calpastatin and various known calpain substrates, such as ataxin-3. A secondary aim of this study was to explore whether short-term treatment with calpain inhibitor compounds could prevent calpain-mediated cleavage and provide therapeutic benefit. We hypothesise that calpain overactivity may be an early disease phenotype, leading to later motor impairments and neuronal loss. Further, we hypothesise that intercepting the onset of calpain overactivity, via treatment with calpain inhibitor compounds, could yield therapeutic benefit in male mice modelling MJD.

## Results

### Phenotype characterisation experiments

#### CMVMJD135 mice develop neurological symptoms from 7 weeks of age

Analysis of body weight progression revealed that MJD mice do not gain weight like littermate controls, with weight gain stagnating from 9 or 10 weeks of age for MJD animals whilst littermate controls continue to gain weight. Two-way repeated measures analysis of weekly body weight measurements revealed a statistically significant effect of genotype (*P* = 0.0367), a statistically significant effect of age (*P* < 0.0001) and a significant genotype x age interaction effect (*P* < 0.0001, Supplementary Fig. 1A). Post-hoc multiple comparisons revealed a statistically significant difference across genotypes at 14 and 15 weeks of age (*P* = 0.0208 and *P* = 0.0112, respectively). CMVMJD135 mice and littermate controls were monitored for neurological symptoms (abnormal hindlimb reflex, tremor and gait abnormalities) weekly as a means of tracking disease progression. CMVMJD135 mice started developing neurological symptoms from as early as 6 weeks of age, whilst wildtype littermate controls remained neurologically healthy for the entire duration of the experiment. Two-way repeated measures analysis of variance (ANOVA) of neurological score revealed a statistically significant effect of genotype (*P* < 0.0001), a statistically significant effect of age (*P* < 0.0001) and a significant genotype x age interaction effect (*P* < 0.0001, Supplementary Fig. 1B). Post-hoc multiple comparisons revealed a statistically significant difference across genotypes, starting at 7 weeks of age. Next, we compared inherited CAG repeat length with neurological score at 12 weeks of age, finding a strong positive correlation (*r* = 0.8486) that was statistically significant (*P* = 0.0038, Supplementary Fig. 1C), suggesting mice with a longer inherited CAG length also displayed a higher neurological score, indicative of more severe neurological symptoms. These findings indicate that CMVMJD135 mice exhibit impaired weight gain compared to littermate controls and a progressive neurological decline correlated with CAG repeat length.

#### Detection of cleaved fragments of αII-spectrin using enzyme-linked immunosorbent assay

**Figure 1:**
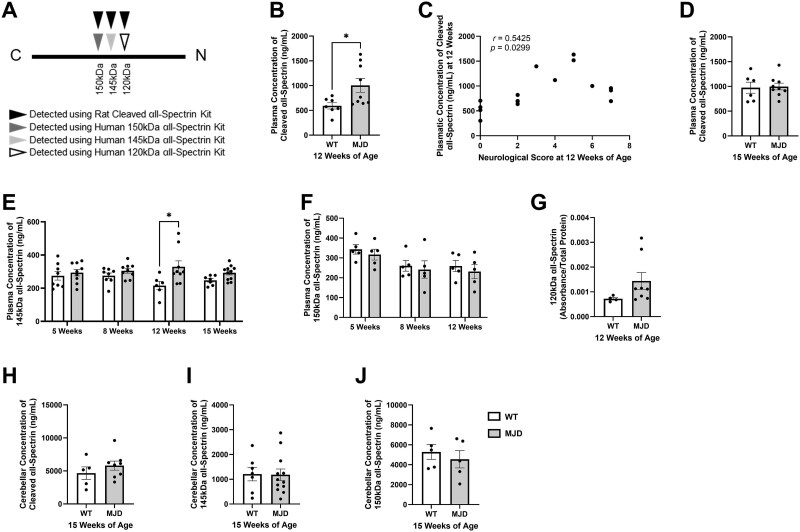
Analysis of the amount of αII-spectrin cleavage products in plasma and cerebellar lysates within the same experimental cohort at different ages. A) Schematic highlighting the detection of different αII-spectrin cleavage fragments via four commercially available ELISA kits. B) The concentration of αII-spectrin cleavage products in 12-week plasma, detected using a rat αII-spectrin ELISA kit. C) Correlational analysis examining plasmatic concentrations of cleaved αII-spectrin and total neurological score at 12 weeks of age. D) The concentration of αII-spectrin cleavage products in 15-week plasma, detected using a rat αII-spectrin ELISA kit. E) Analysis of the plasmatic concentration of 145 kDa αII-spectrin in samples obtained at 5, 8, 12 and 15 weeks of age. F) Analysis of the concentration of 150 kDa αII-spectrin in plasma samples obtained at 5, 8 and 12 weeks of age. G) The relative amount of 120 kDa αII-spectrin in 12-week-old plasma samples, displayed as absorbance relative to total protein due to issues with ELISA kit αII-spectrin standards. H) The concentration of cleaved αII-spectrin in cerebellar lysates detected using a rat ELISA kit (not fragment specific). I) The concentration of calpain-specific 145 kDa αII-spectrin fragment in cerebellar lysates from 15-week-old mice. J) The concentration of 150 kDa αII-spectrin cleavage product in cerebellar lysates from 15-week-old mice. Samples and data obtained from male mice only. Graphs are displaying the group mean ± SEM. ^*^represents *P* < 0.05. *n* = 4–9 per group.

To determine whether calpain activity may be increased in MJD mice and the age at which this might occur, we collected blood samples from the same experimental cohort at four different ages of disease; 5 weeks of age where animals are considered presymptomatic, 8 weeks of age where animals show the earliest signs of neurological symptoms, 12 weeks of age where neurological symptoms are found to be significantly increased in MJD animals relative to wildtype animals and 15 weeks of age. We used four different commercially available enzyme-linked immunosorbent assay (ELISA) kits to determine the concentration of different αII-spectrin cleavage fragments (also known as α-spectrin breakdown products, SBPDs) in plasma (Fig. 1A). First, we used an ELISA kit designed to detect the presence of SBPDs (specific to the rat isoform of αII-spectrin) at ages with established disease symptoms, with the α-spectrin protein sequence highly conserved across mammalian species [47]. Analysis of 12-week plasma samples revealed an increased concentration of cleaved αII-spectrin products, i.e. SBPDs, in MJD plasma samples compared to wildtype plasma samples (*P* = 0.0275, Fig. 1B). Interestingly, the plasmatic concentration of cleaved αII-spectrin detected at 12 weeks of age was found to be positively correlated with total neurological score at 12 weeks of age (*r* = 0.5425, Fig. 1C). This correlation was found to be statistically significant (*P* = 0.0299), suggestive of a relationship between αII-spectrin and neurological symptoms. In contrast, we did not detect any differences in the amount of cleaved αII-spectrin in plasma samples from 15-week-old MJD and wildtype mice (*P* = 0.8515, Fig. 1D).

We hypothesised that detection of non-specific αII-spectrin cleavage fragments, i.e. SBDPs produced by any form of proteolytic cleavage of full-length αII-spectrin, may confound our results as αII-spectrin is also known to be cleaved by calpains and other proteases, such as caspases [33, 48]. Hence, we tested the same plasma samples again using ELISA kits designed to detect αII-spectrin cleavage fragments of certain sizes (human isoform of αII-spectrin) at a range of ages, allowing interrogation of whether the presence of α-spectrin fragments produced by enhanced calpain activity (145 kDa SBDP or 150 kDa SBDP) were increased relative to other α-spectrin fragments which can arise from caspase-mediated cleavage of α-spectrin (150 kDa SBDP or 120 kDa SBDP) [48].

Two-way repeated measures analysis of variance of the concentration of the calpain-specific 145 kDa αII-spectrin fragment (i.e. 145 SBDP) in plasma across different timepoints revealed a statistically significant effect of genotype (*P* = 0.0013, Fig. 1E). However, no statistically significant effects of age or interaction effects were detected (*P* = 0.7630 and *P* = 0.1650, respectively). Tukey’s post-hoc comparisons revealed a statistically significant effect when the wildtype and MJD samples were compared at 12 weeks of age (*P* = 0.0207).

After confirming that the calpain specific 145 kDa fragment of αII-spectrin was increased in MJD animals, we next examined the presence of the 150 kDa αII-spectrin fragment (i.e. 150 SBPD) which can be produced by either calpains or caspases [33, 48]. Two-way repeated measures analysis of variance revealed a statistically significant effect of age on 150 kDa αII-spectrin (*P* = 0.0170, Fig. 1F). However, no effects of genotype or interaction effects were detected (*P* = 0.4455 and *P* = 0.9849, respectively). Due to limitations in total plasma volume obtained, the plasmatic concentration of 150 kDa αII-spectrin could only be determined in samples from 5, 8 and 12 weeks, with insufficient plasma volume remaining to determine 150 kDa αII-spectrin in 15-week plasma samples. We also examined the presence of 120 kDa αII-spectrin (i.e. 120 SBDP), a caspase cleavage product, in 12-week plasma samples only (as this was the only timepoint with sufficient volume remaining) and found no difference in the absorbance relative to total protein concentration between wildtype and MJD plasma samples (*P* = 0.1729, Fig. 1G). Unfortunately, the precise concentration of 120 kDa αII-spectrin could not be determined, due to issues with the protein standards provided with the 120 kDa αII-spectrin ELISA kit, and the samples could not be re-run due to insufficient plasma volume.

Lastly, we examined the concentration of cleaved αII-spectrin in cerebellar lysates from 15-week-old mice. We observed similar levels across wildtype and MJD animals of general cleaved αII-spectrin (*P* = 0.3423, Fig. 1H), calpain-specific 145 kDa SBDP, (*P* = 0.3423, Fig. 1I) and 150 kDa SBDP (*P* = 0.5414, Fig. 1J). To summarise, our ELISA results suggest that plasmatic levels of 145 kDa cleaved αII-spectrin, i.e. 145 SBDP, a fragment produced exclusively by calpain cleavage of αII-spectrin, is increased from 12 weeks of age in CMVMJD135 males when compared to wildtype males.

#### Examination of calpain and Calpastatin levels in cerebellar tissue

**Figure 2:**
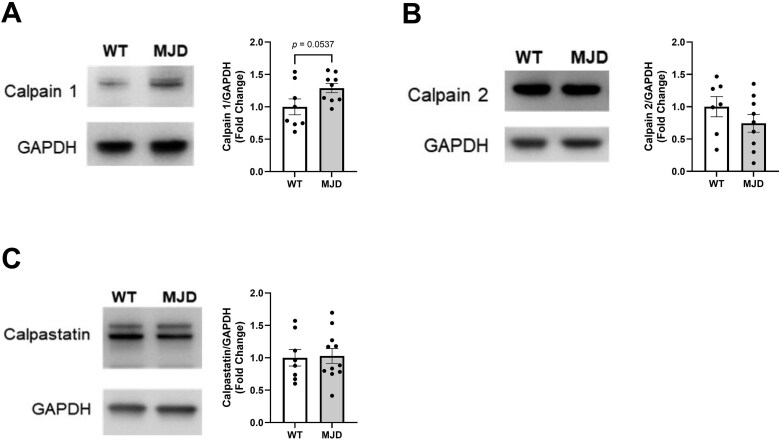
Immunoblot analysis of the calpain system (calpain 1, calpain 2 and calpastatin) in cerebellar protein lysates from 15-week-old mice. Western blotting for calpain 1 revealed the presence of two calpain 1 immunoreactive bands (A), with analysis of the 78 kDa activated/autolysed form of calpain 1 revealed a difference across genotypes that trended towards statistical significance (B). Western blotting for calpain 2 revealed the presence of one immunoreactive band (C) which did not significantly differ across genotype groups (D). Western blotting for calpastatin revealed two immunoreactive bands (E) which did not significantly differ across genotypes (F). *n* = 7–8 wildtype littermate controls, *n* = 8–10 MJD mice expressing human mutant *ATXN3*. Samples and data obtained from male mice only. Graphs are displaying the group mean ± SEM.

Western blot analysis of cerebellar protein lysates from 15-week-old mice expressing human mutant ataxin-3 or wildtype littermate controls revealed the presence of two protein bands immunoreactive for calpain 1 at approximately 80 kDa and 78 kDa (Fig. 2A). The lower molecular weight calpain 1 band is likely the autolysed/activated form of calpain 1 and this band appeared to be more prominent in MJD samples compared with samples from wildtype mice. Densiometric analysis of the lower molecular weight calpain 1 band revealed a trend towards statistical significance across genotypes (*P* = 0.0537, Fig. 2B). Immunoblot analysis of calpain 2 revealed the presence of one distinct protein band (Fig. 2C), which did not significantly differ across genotype groups (*P* = 0.2375, Fig. 2D). We observed two distinct protein bands that were immunoreactive for calpastatin in cerebellar lysates (Fig. 2E), consistent with published reports of multiple calpastatin bands between 130 kDa and 100 kDa [49–51], likely produced by alternative splicing of calpastatin [52]. Densiometric analysis of both calpastatin bands revealed no statistically significant differences across genotypes (*P* = 0.8679, Fig. 2F).

We performed quantitative PCR (qPCR) to assess *Capn1* and *Capn2* gene expression in forebrain tissue from a subset of 15-week-old Cohort 1 animals. *Capn1* expression was found to be similar across genotypes when normalised to the reference genes *Rpl27* and *β2M*, with no statistical significance detected (Supplementary Fig. 2A, B). Similarly, *Capn2* expression was comparable between genotypes when normalised to *Rpl27* and *β2M*, with no statistically significant differences (Supplementary Fig. 2C, D).

#### Examination of calpain substrates in cerebellar tissue

**Figure 3:**
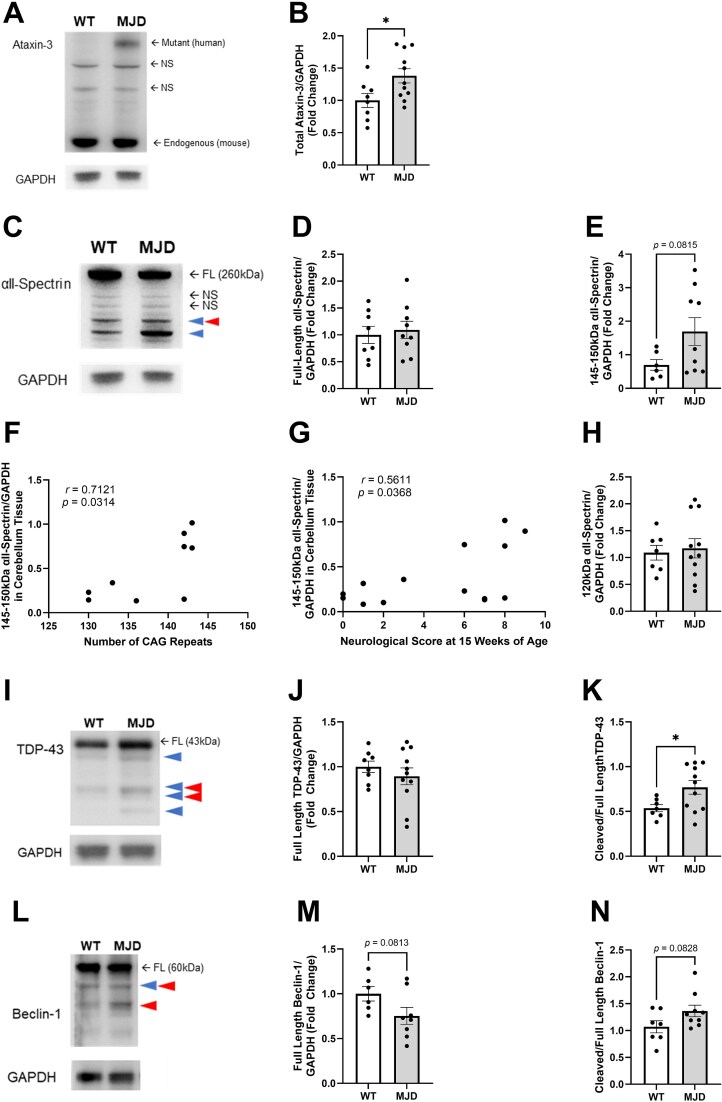
Levels of full-length and cleaved calpain substrates in cerebellar tissue. A) Immunoblotting of ataxin-3 revealed human ataxin-3 and endogenous mouse ataxin-3 but no cleavage fragments were detected. B) Analysis of mutant ataxin-3 levels across genotypes. C) Immunoblotting of αII-spectrin revealed full-length protein and cleavage fragments at 150 and 145 kDa. D) Analysis of full-length α-spectrin. E) Analysis of cleaved (150-145 kDa) αII-spectrin. F) Correlational analysis comparing the presence of cleaved αII-spectrin in cerebellum tissue (15 weeks of age) with inherited CAG length in MJD animals. G) Correlational analysis comparing the presence of cleaved αII-spectrin in cerebellum tissue (15 weeks of age) with total neurological score at 15 weeks of age. H) Immunoblot analysis of 120 kDa cleaved αII-spectrin relative to GAPDH. I) Immunoblotting of TDP-43 revealed full-length protein and cleavage fragments ranging from 40-25 kDa. J) Analysis of full-length TDP-43 levels. K) Analysis of levels of all TDP-43 cleavage fragments across genotypes. L) Immunoblotting of beclin-1 revealed the presence of full-length protein and two previously reported cleavage fragments at 50 kDa and 37 kDa. M) Analysis of full-length beclin-1 levels. J) Analysis of the amount of total cleaved beclin-1 across genotype groups. N) Analysis of cleaved beclin-1 relative to full-length beclin-1. *n* = 7–10 per group, ^*^represents *P* < 0.05, ^***^represents *P* < 0.001. NS indicates non-specific bands. Blue arrowheads represent cleavage fragments produced by calpain cleavage, red arrowheads represent cleavage fragments produced by caspase cleavage. Samples and data obtained from male mice only. Graphs are displaying the group mean ± SEM.

As examination of calpain protein expression levels provides little insight into the proteolytic activity of the calpain system, we next aimed to investigate if cleavage of known calpain substrates was increased in cerebellar tissue from 15-week-old CMVMJD135 mice. First, we examined total levels of ataxin-3 the protein affected in MJD. We observed one band (approximately 75 kDa) that was present in MJD animals that was not present in wildtype littermates which we interpreted to be full-length mutant human ataxin-3 and a strong band in both groups at the expected height of endogenous mouse ataxin-3 (Fig. 3A, see Supplementary Fig. 3A for full image). We observed several non-specific bands in both groups and a distinct lack of bands that could be interpreted as cleaved mutant human ataxin-3. Densiometric analysis of total ataxin-3 intensity levels (i.e. endogenous mouse ataxin-3 and mutant human ataxin-3), relative to GAPDH revealed a statistically significant increase in total ataxin-3 in MJD animals (*P* = 0.0282, Fig. 3B).

Immunoblotting for αII-spectrin revealed the presence of full-length αII-spectrin and two prominent cleavage fragments at 150 kDa and 145 kDa (Fig. 3C, see Supplementary Fig. 3B for full image). Quantification of full-length αII-spectrin did not reveal any statistical differences across genotypes (*P* = 0.6845, Fig. 3D). Densiometric analysis of 145-150 kDa cleaved αII-spectrin (150 and 145 kDa fragments) normalised to GAPDH suggested a trend towards increased αII-spectrin in MJD animals; however, this did not reach statistical significance (*P* = 0.0815, Fig. 3E). To determine if the presence of 145-150 kDa cleaved αII-spectrin was related to abnormal expansion of *ATXN3*, we checked if levels of cleaved αII-spectrin in cerebellum tissue correlated with the number of inherited CAG repeats within *ATXN3*. Pearson’s correlation analysis revealed a moderate positive correlation between 145-150 kDa cleaved αII-spectrin levels (relative to GAPDH) and CAG repeat length (*r* = 0.7121) that was statistically significant (*P* = 0.0314, Fig. 3F). Further, a moderate positive correlation (*r* = 0.5611) was also detected when analysing the relationship between cerebellar levels of 145-150 kDa cleaved αII-spectrin levels (relative to GAPDH) and total neurological score at 15 weeks of age (*P* = 0.0368, Fig. 3G). Levels of 120 kDa cleaved αII-spectrin, produced by caspase cleavage of αII-spectrin [46], in 15-week-old cerebellar lysates were found to be similar across genotypes (*P* = 0.7483, Fig. 3H).

Immunoblotting of mouse TDP-43, a protein known to be aberrantly cleaved by calpains in sporadic and familial motor neuron disease [53] and known to aggregate in MJD [54], revealed the presence of full-length TDP-43 and several cleavage fragments ranging from 40-25 kDa (Fig. 3I, see Supplementary Fig. 3C for full western image). An unpaired t-test was used to compare levels of full-length TDP-43, revealing no statistically significant difference across genotypes (*P* = 0.3970, Fig. 3J). In contrast, the ratio of cleaved to full-length TDP-43 was found to be significantly increased in MJD animals compared to wild type controls (*P* = 0.0358, 3K).

Next, we examined levels of beclin-1, a protein involved in initiation of autophagy, as autophagy impairments have previously been reported in MJD patients [55, 56] and preclinical models of MJD [57]. Immunoblotting revealed the presence of full-length beclin-1 and two additional cleavage fragments at ~ 50 kDa and ~ 37 kDa (Fig. 3L, see Supplementary Fig. 3D). Densiometric analysis of full-length beclin-1 normalised to GAPDH revealed decreased full-length beclin-1 in MJD animals compared to wildtype animals; however, we did not detect a statistical difference (*P* = 0.0813, Fig. 3M). Similarly, analysis of the ratio of all cleaved beclin-1 fragments relative to full-length beclin-1 revealed increased levels of cleaved beclin-1 in MJD animals; however, this did not reach statistical significance (*P* = 0.0828, Fig. 3N).

#### Examination of the calpain system and calpain substrates in brainstem tissue

Next, we explored whether levels of calpain system proteins (calpain 1, calpain 2 and calpastatin) or cleavage of calpain substrates were increased in brainstem (pons and medulla oblongata) protein lysates obtained from the same cohort of experimental animals. Western blotting of brainstem lysates for calpain 1 revealed the presence of multiple bands near 75 kDa, likely full-length and autolysed forms of calpain 1 (Supplementary Fig. 4A). Quantification of calpain 1 relative to GAPDH (Supplementary Fig. 4B) did not significantly differ across wildtype and MJD animals in brainstem lysates (*P* = 0.1838, Supplementary Fig. 4C). Immunoblotting of brainstem lysates for calpain 2 revealed one band near 75 kDa (Supplementary Fig. 4D). Quantification of calpain 2 relative to GAPDH (Supplementary Fig. 4E) revealed similar levels of calpain 2 normalised to GAPDH across genotypes (Supplementary Fig. 4F, *P* = 0.5296). Lastly, western blotting of brainstem lysates for calpastatin revealed the presence of two bands between 100 and 150 kDa (Supplementary Fig. 4G). Quantification of calpastatin levels relative to GAPDH (Supplementary Fig. 4H) were found to be similar in brainstem lysates across wildtype and MJD animals (Supplementary Fig. 4I, *P* = 0.4546).

Immunoblotting of brainstem lysates for ataxin-3 revealed the presence of full-length mutant, human ataxin-3 at ~ 75-80 kDa and endogenous mouse ataxin-3 at ~ 40 kDa, however no ataxin-3 cleavage fragments could be identified (Supplementary Fig. 5A). Quantification of the total ataxin-3 (mutant human ataxin-3 and endogenous mouse ataxin-3) normalised to GAPDH (Supplementary Fig. 5B) revealed significantly increased levels of total ataxin-3 in MJD animals when compared to wildtype animals (*P* = 0.0024, Supplementary Fig. 5C). Further examination of calpain substrates did not reveal any statistically significant differences across genotypes in the amount of full-length or cleaved αII-spectrin (*P* = 0.5143 and *P* = 0.4606 respectively, Supplementary Figs. 5D-G), full-length or cleaved beclin-1 (*P* = 0.3408 and *P* = 0.2381 respectively, Supplementary Figs. 5H-J) or full-length or cleaved TDP-43 (*P* = 0.5632 and *P* = 0.3529 respectively, Supplementary Figs. 5K-N) within brainstem protein lysates. In summary, we did not observe any evidence of heightened calpain activity or calpain-mediated cleavage within brainstem tissue obtained from 15-week-old male CMVMJD135 mice.

#### Mice expressing human mutant ataxin-3 did not display altered levels of AMPA receptor subunits

It has previously been reported that altered expression of α-amino-3-hydroxy-5-methyl-4-isoxazolepropionic acid (AMPA) receptor subunits can increase calcium permeability and lead to downstream calpain activation [58]. To investigate if changes in AMPA receptor subunit levels are contributing to increased calpain activation in CMVMJD135 mice, we immunoblotted cerebellar lysates for GluA1, GluA2, GluA3 and GluA4 from wildtype and MJD mice. There were no statistically significant changes detected across genotypes for any of the investigated receptor subunits (GluA1 *P* = 0.1756, Supplementary Fig. 6A; GluA2 *P* = 0.1105, Supplementary Fig. 6B; GluA3 *P* = 0.9806, Supplementary Fig. 6C; GluA4 *P* = 0.6254, Supplementary Fig. 6D).

### Short-term treatment of MJD mice with calpeptin

#### Daily administration of calpeptin was found to reduce neurological symptoms in MJD animals

**Figure 4:**
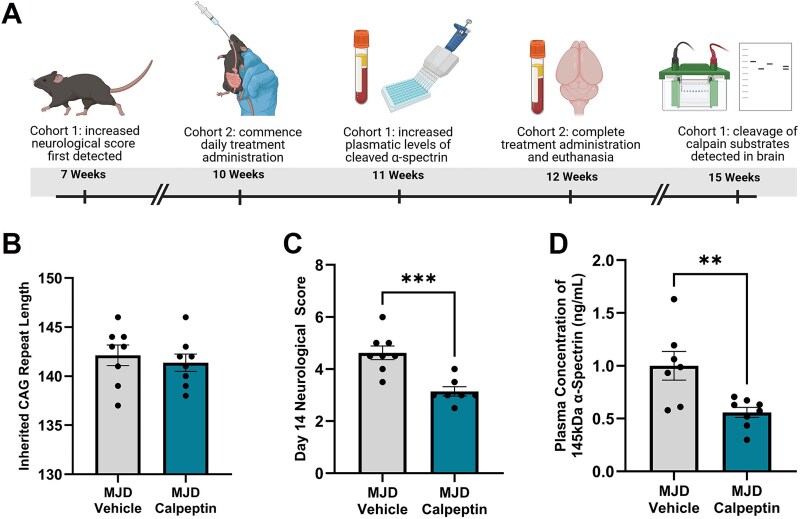
Daily administration of 2 mg/kg calpeptin for a period of 14 days produced positive effects in MJD animals. A) Schematic highlighting the experimental timeline and onset of relevant disease phenotypes. B) Analysis of the inherited CAG repeat length within human *ATXN3* across treatment groups. C) Analysis of day 14 neurological score revealed amelioration of neurological symptoms in calpeptin treated animals. D) The concentration of 145 kDa αII-spectrin, produced exclusively by calpain cleavage, was found to be significantly decreased in calpeptin treated animals. *n* = 8–7 vehicle treated MJD animals, *n* = 7–8 2 mg/kg calpeptin treated MJD animals. ^**^represents *P* < 0.01, ^***^represents *P* < 0.001. Samples and data obtained from male mice only. Graphs are displaying the group mean ± SEM. Figure was created using Biorender.com.

To confirm whether calpain inhibition could offer therapeutic benefit as a treatment for MJD, mice were randomly allocated to one of two experimental groups (oral administration of 2 mg/kg calpeptin or vehicle treatment) and administered treatments daily for a period of 14 days, starting from 10 weeks of age (see Fig. 4A for a summary of the experimental timeline). We first confirmed the CAG repeat length within inherited human *ATXN3* was similar across treatment groups (*P* = 0.5921, Fig. 4B), confirming any phenotype differences observed were an effect of treatment. Comparison of the total neurological score following 14 days of daily treatment of the treatment study revealed a statistically significant decrease in total neurological score in calpeptin treated animals when compared to vehicle treated animals (*P* < 0.0006, Fig. 4C), suggesting less severe neurological symptoms. The concentration of 145 kDa αII-spectrin, a fragment produced exclusively by calpain-mediated cleavage of αII-spectrin, was also found to be significantly decreased within plasma samples obtained on day 14 from calpeptin treated MJD animals when compared to vehicle treated MJD animals (*P* = 0.0064, Fig. 4D), demonstrating that orally administered 2 mg/kg calpeptin yielded successful target engagement.

#### Calpain inhibitor treatments altered levels of autolysed calpain 1 and Calpastatin

**Figure 5:**
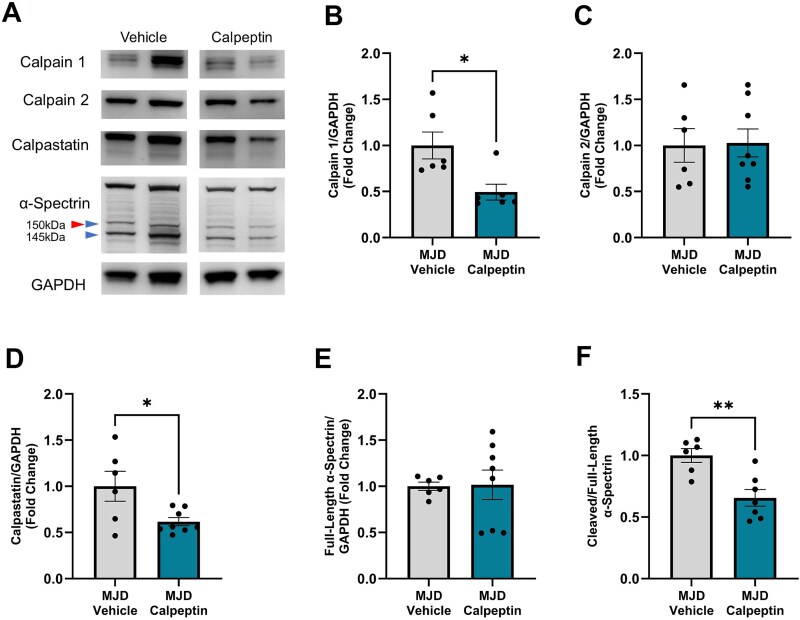
Daily administration of 2 mg/kg calpeptin altered protein expression of calpain system proteins and the presence of cleaved αII-spectrin within cerebellar lysates*.* A) Representative western blots displaying protein levels of calpain 1, calpain 2, calpastatin and αII-spectrin*.* B) Protein levels of calpain 1 were found to be significantly decreased following daily treatment with 2 mg/kg calpeptin when compared to vehicle treatment. C) Calpeptin treatment did not alter protein expression of calpain 2. D) Daily treatment with 2 mg/kg calpeptin decreased protein levels of calpastatin when compared to vehicle treatment. E) Protein levels of full-length αII-spectrin were similar across cerebellar protein lysates from vehicle treated and calpeptin treated animals. F) Levels of cleaved αII-spectrin relative to full-length αII-spectrin were found to be decreased in calpeptin treated MJD animals, when compared to vehicle treated MJD animals. *n* = 6 vehicle treated MJD animals, *n* = 6–8 2 mg/kg calpeptin treated MJD animals. ^*^represents *P* < 0.05, ^**^represents *P* < 0.01. Blue arrowheads represent cleavage fragments produced by calpain cleavage, red arrowheads represent cleavage fragments produced by caspase cleavage. Samples and data obtained from male mice only. Graphs are displaying the group mean ± SEM.

To examine the effect of calpain inhibitor treatments on the calpain system, we examined the levels of calpain 1, calpain 2, calpastatin and αII-spectrin in cerebellar lysates harvested after two weeks of treatment (Fig. 5A). Densiometric analysis of calpain 1 in cerebellar lysates revealed the presence of three bands as described previously; 80 kDa inactive calpain 1, 78 kDa autolysed/activated calpain 1 and 76 kDa autolysed/inactive calpain 1. Calpain 1 levels relative to GAPDH revealed were found to be significantly reduced in calpeptin treated MJD animals when compared to vehicle treated animals (*P* = 0.0134, Fig. 5B). Immunoblotting of cerebellar lysates for calpain 2 revealed one clear band at the expected height of full-length calpain 2. We detected no change in the levels of calpain 2 across treatment groups (*P* = 0.9111, Fig. 5C). Immunoblotting cerebellar lysates for calpastatin revealed the presence of one distinct band with another fainter band, which could indicate alternative splicing of calpastatin. Similarly to calpain 1, short-term daily administration of calpeptin decreased calpastatin protein levels (relative to GAPDH) when compared to vehicle treatment (*P* = 0.0236, Fig. 5D).

#### Effect of short-term treatment with calpain inhibitors on calpain substrates

To complement examination of calpain expression levels, we also examined the presence of calpain-mediated cleavage products in cerebellar lysates using western blotting to further elucidate calpain activity. Considering our finding of decreased plasmatic concentration of 145 kDa αII-spectrin following calpeptin treatment, we first examined whether protein levels of αII-spectrin were affected in cerebellar protein lysates. Immunoblotting of cerebellar lysates for αII-spectrin revealed the presence of full-length αII-spectrin and two distinct cleavage fragments at 150 kDa and 145 kDa (Fig. 5A, see Supplementary Fig. 6A for full western image). Densiometric analysis of full-length αII-spectrin did not reveal any statistically significant changes across treatment groups (*P* = 0.9347, Fig. 5E). In contrast, levels of cleaved αII-spectrin relative to full-length αII-spectrin were found to be significantly decreased in calpeptin treated MJD animals when compared to vehicle treated MJD animals (*P* = 0.0027, Fig. 5F).

Next, we examined levels of calpain substrates ataxin-3, TDP-43 and beclin-1 in cerebellar lysates. Consistent with our previous findings, we did not observe any ataxin-3 positive bands that could indicate cleavage of ataxin-3 in CMVMJD135 mice (Supplementary Fig. 7B-C). Irrespective of ataxin-3 cleavage, previous work from our laboratory has shown that treatment with calpeptin or BLD-2736 can induce autophagic degradation of full-length mutant ataxin-3 in transgenic MJD zebrafish [43, 59, 60], thus we checked for similar treatment effects following short-term treatment in MJD mice. We did not observe any treatment effects on full-length ataxin-3 (*P* = 0.3554, Supplementary Fig. 6D).

Next, we examined calpain-mediated cleavage of TDP-43. Immunoblotting of cerebellar lysates for TDP-43 revealed the presence of full-length TDP-43 and three distinct cleavage fragments (Supplementary Fig. 7E-F). We did not detect any effect of calpain inhibitor treatments on full-length TDP-43 (*P* = 0.4699, Supplementary Fig. 7G) or TDP-43 cleavage fragments (*P* = 0.6664, Supplementary Fig. 7H).

Lastly, we examined the effects of calpain inhibitor treatments on calpain-mediated cleavage of beclin-1. Immunoblotting of cerebellar lysates for beclin-1 revealed full-length beclin-1 and two cleavage fragments at 50 kDa and 37 kDa (Supplementary Fig. 7I-J). Short-term daily treatment with calpeptin did not alter levels of full-length beclin-1 (*P* = 0.7984, Supplementary Fig. 7K) or beclin-1 cleavage fragments (*P* = 0.7304, Supplementary Fig. 7L).

## Discussion

### Summary of longitudinal plasma findings

In this study, we aimed to explore whether the calpain system was dysregulated in transgenic CMVMJD135 mice modelling MJD and to identify precisely when calpain activity may be increased through examination of cleaved αII-spectrin in longitudinal plasma samples. Using four different commercially available ELISA kits aimed at detecting different αII-spectrin cleavage fragments, we detected increased presence of cleaved αII-spectrin (non-specific and 145 kDa, i.e. 145 SBDP) in plasma from 12-week-old MJD mice. Further, we observed a positive correlation between plasmatic levels of total cleaved αII-spectrin and neurological score at 12 weeks of age, highlighting a relationship between the presence of cleaved αII-spectrin and neurological impairments. To our knowledge, this is the first report of increased levels of cleaved αII-spectrin (i.e. SBDPs) in plasma from a neurodegenerative disease mouse model, highlighting that this biomarker is not specific to brain injury models but could be used as a more general marker of calpain overactivity. Further, we did not detect any statistically significant differences in the plasmatic levels of 150 kDa (i.e. 150 SBDP) or 120 kDa cleaved αII-spectrin (i.e. 120 SBDP), fragments that can be produced by activation of caspase proteases [33], at any of the timepoints examined. However, interpretation of the underlying mechanistic cause of αII-spectrin cleavage or breakdown cannot be determined when using non-specific cleaved αII-spectrin ELISA kit, such as the rat ELISA kit developed by MyBioSource (catalogue *#* MBS380894). Our study is strengthened by the utility of multiple ELISA kits designed to detect specific αII-spectrin fragments, also known as SBDPs, as we are able to pinpoint precisely which SBDP product is increased and attribute this increased cleavage to a specific protease. Collectively, our plasma findings highlight specific overactivation of calpain proteases in CMVMJD135 mice, aligning with a wealth of literature highlighting calpains as the pathogenic protease in MJD [24, 41–45]. The findings from this study highlight that increased calpain-mediated cleavage of αII-spectrin aligns with the onset of neurological symptoms in male CMVMJD135 transgenic mice and thus is associated with MJD pathogenesis. Future studies should seek to examine whether cleaved αII-spectrin can be detected in plasma or CSF samples from MJD patients and determine whether the presence of increased cleaved αII-spectrin correlates with neurological symptom onset or severity. If plasmatic levels of cleaved αII-spectrin are found to align with disease progression or severity in MJD patients, this biomarker could be a powerful tool for future clinical trials and could aid in patient stratification.

### Calpain overactivity phenotype characterisation in cerebellar tissue

Next, we aimed to explore whether the calpain system was dysregulated in the cerebellum, through examination of levels of calpain 1, calpain 2 and calpastatin in post-mortem cerebellar tissue from 15-week-old mice. We detected a trend towards increased protein levels of calpain 1 in the cerebellum, but no genotype differences in levels of calpain 2 or calpastatin. We also analysed gene expression levels of *Capn1* and *Capn2* in a subset of the same experimental animals, finding no genotype differences in gene expression. This finding suggests that any changes in calpain 1 protein expression or activity cannot be attributed to underlying transcriptional differences at the gene level. However, protein levels are not reflective of enzyme activity and thus must be interpreted with caution. Immunoblotting revealed two distinct calpastatin bands in our cerebellar lysates, possibly produced due to alternative splicing of calpastatin. It is possible that analysis of two isoforms together may have confounded our results, reducing the ability to detect genotype differences. Our finding of no genotype difference in calpastatin levels contrasts with previous findings from Simões *et al* [45] who reported decreased levels of calpastatin in a lentiviral mouse model expressing *ATXN3* with 72 CAG repeats and brain tissue from three MJD patients. Simões *et al* [45] hypothesised that mutant expansion of ataxin-3 may interact with calpastatin, leading to downregulation; however, our findings directly refute this hypothesis. The lack of downregulation of calpastatin in 15-week-old male CMVMJD135 mice in the present study suggests that there may be an alternative underlying mechanistic cause of calpain overactivation separate to any change in calpastatin protein expression or activity. The present study has not explored whether the activity of the calpastatin protein is affected in CMVMJD135 mice; it is possible that calpastatin activity, and the endogenous inhibition of calpains, could be affected despite a lack of effect observed in calpastatin protein levels. Further work is required to fully understand the mechanisms underlying calpastatin dysregulation in CMVMJD135 mice and MJD more broadly.

Considering that protein levels of calpain 1 and calpain 2 do not reflect protease activity, we next examined whether known calpain substrates were cleaved in CMVMJD135 mice. Whilst we observed one ataxin-3 positive band at around 75 kDa, aligning with previous reports of mutant, expanded ataxin-3 in CMVMJD135 mice [61, 62], we did not observe any other bands that could be indicative of cleaved mutant ataxin-3. Considering that CMVMJD135 mice develop many symptoms akin to clinical MJD without detection of ataxin-3 cleavage, we hypothesise that calpain-mediated cleavage is not critical to initiate the onset of the neurodegenerative process in CMVMJD135 mice. This finding is interesting as it directly opposes the toxic fragment hypothesis that suggests that proteolytic cleavage of disease-causing proteins initiates a toxic cascade of events, leading to neurodegeneration [63, 64]. One factor to consider is that CMVMJD135 mice are not an overexpression model and were in fact designed to express mutant *ATXN3* at near endogenous levels [62]. Our immunoblotting results suggest that endogenous mouse ataxin-3 is more strongly expressed than human mutant ataxin-3. Considering cleavage fragments are likely to be more weakly expressed than full-length protein, it is possible that mutant human ataxin-3 cleavage fragments may not be detectable via western blotting and may require more sensitive methodologies such as qPCR or mass spectrometry. Interestingly, Hübener *et al* [24] reported that ataxin-3 may be more efficiently cleaved by calpain 2 than calpain 1. It is possible that the lack of ataxin-3 cleavage observed in this study could be caused by lack of calpain 2 activity, and that investigation of ataxin-3 cleavage at later timepoints, when calpain 2 may be more active, or in other regions of the brain may more insights.

We detected increased cleavage of TDP-43 in cerebellar lysates from 15-week-old MJD mice. To our knowledge, this is the first report of TDP-43 cleavage in any model of MJD. TDP-43, an RNA binding protein, is a known calpain substrate [32] and calpain-mediated cleavage of TDP-43 has been implicated in the pathogenesis of motor neuron disease [53], thus it is plausible that calpain-mediated cleavage of TDP-43 could be a disease mechanism underlying many neurodegenerative diseases. It is also worth noting that TDP-43 protein aggregates have been detected in MJD patient tissue [54], suggesting there may be a link between calpain-mediated cleavage of TDP-43 and TDP-43 neuropathology in MJD.

Further, we detected trends toward increased cleaved αII-spectrin and increased cleaved beclin-1 in MJD mice; however, these comparisons did not reach statistical significance. Future investigations are required to identify whether enhanced calpain activity is present in older CMVMJD135 animals and whether the relative amount of calpain activity correlates with disease progression.

### Lack of calpain-mediated cleavage within brainstem tissue from CMVMJD135

In contrast to our findings from the cerebellum, we did not observe any evidence of calpain system changes or increased calpain-mediated cleavage within brainstem lysates from CMVMJD135 mice when compared to wildtype littermate controls. This finding is surprising, as a previous study from our laboratory identified an increased burden of ataxin-3 aggregates within the medulla oblongata relative to the cerebellum in male CMVMJD135 mice [61], and thus we hypothesised that calpain activity may be heightened in the brainstem relative to the cerebellum. It is possible that this regional difference in calpain activity could be due to regional differences in calcium signalling or regulation, however further studies are required to confirm the underlying cause of this regional difference and identify whether calpain activity within the brainstem could occur at later stages of disease, after the onset of calpain overactivity within the cerebellum.

### What mechanism may contribute to calpain activity in cerebellum of CMVMJD135 mice?

Considering calpastatin was unchanged in CMVMJD135 mice, we hypothesise that changes in calpain activity within the cerebellum are not due to decreased calpastatin causing disinhibition of the calpain system, but perhaps due to an alternative mechanism that may increase calcium signalling and lead to subsequent calpain activity. As calpains are calcium-activated proteases, we hypothesised that calcium entry into cerebellar neurons may be increased, leading to increased calpain activity. One mechanism that has been identified to contribute to calcium dysregulation and downstream calpain activity in neurodegenerative diseases is altered expression of AMPA receptor subunits [65, 66]. However, we detected no genotype differences in expression levels of GluA1, GluA2, GluA3 or GluA4 within the cerebellum. This study did not examine whether levels of unedited GluA2 were altered across genotypes, a factor that could contribute to calcium entry and downstream calpain activity. Whilst not within the scope of this study, exploration of genotype differences in levels of unedited GluA2 within the cerebellum could be explored in future studies. Potential contribution of other calcium receptors, such as ionotropic N-methyl-D-aspartate (NMDA) receptors or voltage-gated calcium channels, should also be investigated in future studies.

### Short-term treatment with calpeptin reduced neurological symptoms and plasmatic levels of 145 kDa αII-spectrin in MJD mice

In previous work from our laboratory, we have identified that treatment with pan calpain inhibitor compound calpeptin provided therapeutic benefit in our transgenic zebrafish model of MJD, including improved swimming performance, reduced cleavage of ataxin-3 and autophagic degradation of ataxin-3 aggregates [43, 60]. In this study, we explored the therapeutic potential of calpeptin treatment in male CMVMJD135 mice modelling MJD. We found that daily treatment of MJD mice with 2 mg/kg calpeptin from 10 to 12 weeks of age (daily treatment for a total of 14 days) was sufficient to produce a statistically significant reduction in neurological score when compared to vehicle treated MJD mice. As increased neurological score is suggestive of worse neurological symptoms and impairments, this finding suggests that calpeptin may ameliorate or slow the development of neurological symptoms. This difference in neurological symptoms cannot be attributed to an underlying difference in inherited CAG repeat length within *ATXN3*, as the inherited CAG repeat length was found to be similar across treatment groups.

In addition to the reduction in neurological score, we also observed a statistically significant decreased in plasmatic concentration of 145 kDa αII-spectrin in calpeptin treated MJD mice when compared to vehicle treated mice. This finding first highlights direct target engagement, i.e. the capability of calpeptin treatment to reduce calpain activity *in vivo*, and secondly, provides exciting evidence that plasmatic levels of 145 kDa cleaved αII-spectrin can be used as a relatively non-invasive measure of calpain-mediated proteolysis, aiding in early assessment of drug treatment efficacy in future studies in CMVMJD135 mice.

### Short-term treatment with calpeptin altered the calpain system within the cerebellum

Following the improvement in neurological score and plasmatic levels of cleaved αII-spectrin, we next investigated the effect of calpain inhibitor treatments on levels of calpains or calpain substrates via western blotting of post-mortem cerebellar tissue. We chose to investigate the effect of calpain inhibitor treatment on calpain-mediated cleavage within the cerebellum, as this is an area of the brain that harbours ataxin-3 aggregates in both MJD patients [20] and CMVMJD135 animals [61]. Our western blotting results revealed that short-term (14 day) treatment with calpeptin was sufficient to produce decreased levels of activated calpain 1, decrease calpastatin levels and decrease the presence of cleaved αII-spectrin fragment, a fragment produced exclusively by calpain cleavage of full-length (260 kDa) αII-spectrin [33]. Collectively, these results suggest that oral administration of 2 mg/kg calpeptin can cross the blood–brain-barrier and inhibit the activity of calpains, and consequent calpain-mediated cleavage, within the brain. Our finding of decreased 145 kDa αII-spectrin within plasma and cerebellar protein lysates following short-term treatment with calpeptin aligns with findings from de la Fuente *et al* who observed decreased presence of 150/145 kDa α-spectrin (also named α-fodrin) in spinal cord tissue from mice modelling spinal muscular atrophy following eight days of treatment with calpeptin [72].

In contrast, short-term daily treatment with 2 mg/kg calpeptin was not found to alter protein levels of mutant human ataxin-3, endogenous TDP-43 or beclin-1 within cerebellar lysates. Proteins such as TDP-43 and beclin-1 are also known to be cleaved by other proteases, such as caspase 3 [73, 74]. Interrogation of calpain-specific and caspase-specific cleavage of TDP-43 and beclin-1 is complicated by the fact that these proteases can produce fragments of equal molecular weight (see coloured arrowheads in Figs. 3 and 5). Considering caspase 3 can be cleaved by calpains [75–77], overactivation of calpains in CMVMJD135 mice may also indirectly trigger increased activation of caspase 3, contributing to the increased fragmentation of substrates such as TDP-43 and beclin-1. We have not examined whether 2 mg/kg calpeptin can reduce caspase-mediated cleavage within the present study, and further experimentation is required to further understand the lack of effect observed on TDP-43 and beclin-1 substrates. Previous studies from our laboratory have suggested that calpeptin treatment may induce autophagic flux in transgenic MJD zebrafish, leading to reduced levels of mutant, expanded ataxin-3 [43]. It is possible that whilst the administered dose, 2 mg/kg, was effective in reducing the plasmatic concentration of 145 kDa cleaved αII-spectrin and calpain 1 levels, this dose may have been suboptimal for autophagy induction. Further, longer treatment duration or different treatment strategies may be required to effectively inhibit the cleavage of TDP-43 or beclin-1. Future studies could perform more thorough treatment comparisons to investigate the optimal treatment strategy for preventing the cleavage of these protein substrates, which are known to be cleaved by both calpains and other proteases such as caspases [33].

### Limitations and future directions

Despite findings of increased calpain-mediated cleavage of αII-spectrin in the plasma of MJD mice at 12- and 15-weeks-old, we observed only minimal signs of increased calpain activity within the MJD cerebellum and no evidence of heightened calpain activity within the MJD brainstem at 15 weeks of age. This finding was surprising considering that the cerebellum is only moderately affected in CMVMJD135 mice, displaying relatively less ataxin-3 protein aggregates and neuronal loss than other regions, such as the pons and medulla [61]. Further, whilst calpain 1 and 2 are expressed within the cerebellum (www.brain-map.org), calpain 1 is more highly expressed in the pons and medulla (www.brain-map.org), corresponding with areas of high ataxin-3 aggregate load [61]. It is possible that other mechanisms, such as regulation of calcium directly within the cerebellum, could explain this regional difference and that the brainstem may be affected at later stages of disease.

A second major limitation of this study was that calpain activity was only explored in male mice in the early stages of disease. We chose to first examine the presence of this phenotype in male mice as first pass, as previous studies from our laboratory have demonstrated that male CMVMJD135 mice follow a more rapid and severe disease course than female CMVMJD135 mice [46]. Follow up studies could explore whether calpain overactivity is also present in female CMVMJD135 mice and whether this phenotype also has a delayed onset in females. Interestingly, in preclinical mouse models of traumatic brain injury, female mice display some level of neuroprotection, with more delayed onset of calpain activation in female mice following diffuse brain injury [78]. Thus, it is plausible that differences in the onset of calpain activity in CMVMJD135 could partly explain the delayed onset of neurological symptoms [46].

Lastly, there was variability in the inheritance of mutant *ATXN3*, with animals in this study expressing mutant human *ATXN3* with CAG lengths ranging from 130 to 146 repeats. It is well reported that increased inherited CAG repeat length is associated with earlier disease onset and more severe disease progression in clinical MJD cohorts [10, 12, 13]. Indeed, we observed a moderate, statistically significant correlation between inherited CAG repeat length and neurological score at 12 weeks of age (Fig. 1C), suggesting that inherited CAG length may also influence disease progression and severity in CMVMJD135 mice. Further, we also observed a moderate statistically significant correlation between cleaved αII-spectrin in the cerebellum and inherited CAG repeat length (Fig. 4F). This suggests that calpain-mediated cleavage of αII-spectrin may occur earlier in animals with a longer inherited CAG length, suggesting earlier onset of this phenotype in higher CAG animals. It has previously been reported that a similar transgenic line expressing mutant human *ATXN3* with 83 CAG repeats under a CMV promoter did not develop MJD phenotypes [79], suggesting CAG repeat thresholds may exist similar to in clinical MJD populations [10–12]. Other studies using this transgenic line have restricted CAG lengths to a minimum of 133, reducing phenotypic variability within experimental cohorts [80–82]. Thus, it is possible that inclusion of such broad range of CAG lengths within the study could increase the phenotypic variability and decrease the effect sizes. Future experiments could seek to clarify whether calpains are overactive from as early as 12 weeks of age in male CMVMJD135 mice expressing *ATXN3* with more than 145 CAG repeats and elucidate the influence inherited CAG length on the onset of aberrant calpain activity in MJD patients.

In summary, this study demonstrates that the calpain system may be dysregulated in male CMVMJD135 mice from as early as 12 weeks of age. We report for the first time that cleaved αII-spectrin, a surrogate of calpain activity, can be detected in plasma samples from male CMVMJD135 mice modelling MJD, providing a relatively non-invasive biomarker of calpain activity that can be correlated with the inherited CAG repeat length and neurological impairments. Irrespective of whether calpain overactivity is a cause or consequence of MJD pathogenesis, our finding of increased cleavage of αII-spectrin in MJD plasma is particularly useful, as plasma is a relatively non-invasive sample that can be easily obtained, and could act as important marker of target engagement (i.e. calpain activity) in future preclinical studies exploring biomarkers and therapeutic potential of calpain inhibition for the treatment of MJD. Our findings suggest that treatment with the calpain inhibitor compound calpeptin may provide therapeutic benefit in mice modelling MJD, however further investigation is needed particularly into the safety of long-term calpain inhibition due to the broad effects such treatment may have.

## Materials and methods

### Experimental animals

This study utilised male CMVMJD135 mice expressing mutant human *ATXN3* under a CMV promoter or male non-transgenic littermates. Cohort 1 (phenotype characterisation experiments) used *n* = 20 male mice (*n* = 12 transgenic mice expressing mutant human *ATXN3* with 130–148 repeats, *n* = 8 wildtype littermates). Cohort 2 (calpain inhibitor treatment experiments) used *n* = 16 male mice (with an inherited CAG repeat length of 137–146, *n* = 8 per treatment). Expression of CMV was confirmed by extraction of DNA from tail tissue (collected at 12–20 days of age) and PCR. Mice were bred at Australian BioResources and transported to Macquarie University. Mice were group housed in individually ventilated cages with littermates for the duration of the experiment. Mice were provided *ad libitum* access to standard chow, water and environmental enrichment and were housed in temperature and humidity-controlled rooms with a 12-hour light/dark cycle (lights on at 6 a.m.). Mice were given a minimum of 5 days to acclimate after arriving at Macquarie University before commencing experimentation (5 weeks of age for phenotype Cohort 1, 10 weeks of age for Cohort 2). All experimental procedures were approved by the Macquarie University Animal Ethics Committee (Animal Research Authority: 2020/028) and were performed in accordance with the Australian Code of Practice for the Care and Use of Animals for Scientific Purposes.

### Neurological monitoring

Mice underwent weekly welfare and neurological monitoring, including assessment of neurological symptoms such as abnormal hindlimb reflex, tremor and ataxic gait on a 4-point scale, where a score of 0 indicated no neurological symptoms and a score of 4 indicated significant neurological impairment. Hindlimb reflex was assessed by briefly suspending the animal by the tail. Animals were given a score of 0 if both hindlimbs were splayed wide when suspended, a score of 1 if there was intermittent partial collapse towards the midline, a score of 2 if one hindlimb was retracted whilst the other hindlimb was splayed wide, a score of 3 if both hindlimbs were fully retracted when suspended or a score of 4 if both hindlimbs indicated paralysis. Tremor was assessed by observation or gently holding enclosed within two hands to feel for tremor. Mice were given a score of 0 if no tremor was present, a score of 1 for a slight, intermittent tremor, a score of 2 for an intermittent but visible tremor, a score of 3 for a continuous, pronounced tremor and a score of 4 if continuous pronounced tremor was found to negatively impact basal activity. Lastly, ataxic gait was assessed by visual observation of an animal’s movement. A score of 0 was given for normal gait, a score of 1 was given for slowed gait, a score of 2 was given for obvious gait abnormalities such as toe walking, foot dragging or hopping, a score of 3 was awarded for lack of hindlimb use for forward motion and a score of 4 was given for no forward motion. The scores for each symptom were totalled, with the highest possible score of 12. Neurological monitoring was performed by the same experimenter throughout the duration of the experiment.

### Plasma collection

Mice were placed in a warming chamber (set at 31°C) or placed under an infrared heat lamp for a minimum of 15 minutes. Mice were restrained within a plastic tube with one hind leg stretched out. The hind leg was shaved and the leg cleaned with 80% ethanol. The saphenous vein was gently pierced with a 24G needle and blood was collected into heparinised capillary tubes. A maximum of 200 μL of whole blood was gently combined with ETDA (62.5 μM) and blood tubes were stored on ice until centrifugation. Tubes were then centrifuged at 5000xg for 12 minutes at 4°C. After centrifugation, the plasma supernatant was collected and aliquoted into a fresh tube and stored at -80°C until experimentation. Protein concentration in plasma samples was measured using a NanoDrop™ 2000 spectrophotometer (Thermo Fisher).

### ELISA quantification of cleaved α-spectrin

To examine the presence of cleaved αII-spectrin, also known as α-spectrin breakdown products (SBDPs), we used three different commercially available enzyme-linked immunosorbent assay (ELISA) kits purchased from MyBioSource (MBS3808941 designed to detect all forms of cleaved αII-spectrin in rat samples, MBS7606326 designed to specifically detect the 150 kDa form of cleaved αII-spectrin, i.e. 150 kDa SBDP in human samples and MBS770193 designed to specifically detect the 145 kDa form of cleaved αII-spectrin in human samples (145 kDa SBPD) and one kit from Xpressbio designed to detect the 120 kDa form of cleaved αII-spectrin in human samples (120 SBDP, catalogue # XPEH4242). Plasma samples were diluted at a concentration of 1:5–1:25, cerebellum samples were diluted at a concentration of 1:100. ELISAs were performed in accordance with kit instructions. The detected concentration of cleaved αII-spectrin was normalised against total protein concentration.

### Calpain inhibitor preparation and administration

Mice expressing mutant human ataxin-3 were randomly allocated to one of two groups; vehicle treatment (0.5% methyl cellulose) or 2 mg/kg calpeptin treatment (Caymen Chemicals #14593) dissolved in 0.5% methyl cellulose. The dose of calpeptin was selected from publications reporting successful inhibition of calpains in rodent models [83–85]. Animals were administered drug treatments once daily via oral gavage, for 14 days. Treatments were administered at a volume of 10 mL/kg according to body weight.

### Euthanasia and tissue collection

For Cohort 1, euthanasia was performed after detecting increased cleaved αII-spectrin in plasma at two timepoints. For Cohort 2, mice were euthanised after 14 days of treatment following 75 minutes after treatment administration. Animals were euthanised via overdose of sodium pentabarbitone (300 mg/kg, i.p.). Once deeply anaesthetised, animals underwent intracardiac perfusion with ice cold 0.9% saline. Brains were harvested and immediately snap frozen in liquid nitrogen, then stored at -80°C until protein extraction.

### Protein extraction

In this study, we chose the cerebellum and brainstem as our regions of interest as these brain regions have been shown to undergo neurodegeneration [3, 20] and contain cleaved ataxin-3 in MJD patients [40], undergo neurodegeneration in male CMVMJD135 mice [61, 86] and expresses both calpain 1 and 2 [87]. Frozen cerebellum (Cohort 1 and Cohort 2) or brainstem (Cohort 1) tissue was dissected. Tissue dedicated for use in ELISAs was homogenised in 0.1 M phosphate buffered saline contained 0.2% Tween-20 containing protease and phosphatase inhibitors (5 μL per mg of tissue). Tissue dedicated for western blotting was in RIPA buffer containing protease and phosphatase inhibitors (5 μL per mg of tissue). Tissue was then sonicated using an Omniruptor 250 Ultrasonic Homogeniser (Omni International). Protein lysates were centrifuged at 13200 x *g* for 20 minutes at 4°C. After centrifugation, the clear supernatant was collected, and protein concentration was determined using a Pierce BCA Protein Assay Kit (Thermo Fisher Scientific).

### Western blotting

Equal amounts of protein were prepared in Laemelli buffer (Bio-Rad) containing NuPage reducing agent (Life Technologies). Proteins were denatured by heating for 5 minutes at 95°C then separated on NuPage 4–12% Bis-Tris gels (Thermo Fisher Scientific) through SDS-PAGE. Proteins were then transferred onto a PVDF membrane (0.45 μm pore size, Merck Millipore) for immunoblot probing. Immunoblots were probed with primary antibodies including rabbit anti-ataxin-3 (1:50000 dilution, gift provided by H. Paulson), mouse anti-alpha spectrin II (C-3) (1:250 dilution, catalogue # sc-48 382, Santa Cruz), rabbit anti-calpain 1 (1:1000 dilution, catalogue #2556S, Cell Signaling Technology), rabbit anti-calpain 2 (1:2000 dilution, catalogue #2539S, Cell Signaling Technology), rabbit anti-calpastatin (1:2000 dilution, catalogue #4146, Cell Signaling Technology), rabbit anti-TDP-43 (1:1000 dilution, catalogue #10782–2-AP, Proteintech), rabbit anti-beclin-1 (1:2500 dilution, catalogue #11306–1-AP, Proteintech), rabbit anti-GFAP (1:1000 dilution, catalogue #, Abcam), mouse anti-neurofilament light chain (1:500 dilution, catalogue #sc-20 012, Santa Cruz), rabbit anti-GluA1 (1:1000 dilution, catalogue #13185, Cell Signaling Technology), rabbit anti-GluA2 (1:1000 dilution, catalogue #AB1768-I, Millipore), rabbit anti-GluA3 (1:1000 dilution, catalogue #4676, Cell Signaling Technology), rabbit anti-GluA4 (1:1000 dilution, catalogue #8070, Cell Signaling Technology) and mouse anti-GAPDH (1:5000 dilution, catalogue #60004–1-Ig, Proteintech). Immunoblots were then probed with appropriate horse-radish peroxidase secondary antibodies (anti-mouse HRP, catalogue #W4028, anti-rabbit HRP, catalogue #W4018 Promega) and visualized via chemiluminescence (normal ECL or SuperSignal West Femto Maximum Sensitivity Substrate, Thermo Fisher). Images were acquired using an ImageQuantLAS400. Band densiometry was quantified using Image Studio Lite and target proteins were normalized against GAPDH or to itself (cleaved/full length). Between probings, immunoblots were stripped using Restore™ PLUS Western blot stripping buffer (catalogue number #46430, Thermo Fisher).

### RNA extraction, cDNA synthesis and qPCR

To assess whether the changes in calpain-mediated cleavage could be due to underlying genotype differences in *Capn1* or *Capn2* gene expression, we performed RT-qPCR on forebrain tissue samples from 15-week-old male mice (Cohort 1—phenotype characterisation cohort, *n* = 5 CMVMJD135 animals and *n* = 5 wildtype littermates). 50 mg of frozen forebrain tissue were suspended in TRIzol™ Reagent (Invitrogen, catalogue # 15 596 026) and homogenised using an Omniruptor 250 Ultrasonic Homogeniser (Omni International). Homogenised tissue samples were centrifuged at 13000 rpm for 15 min, and the supernatant transferred to a new tube. Total RNA was isolated following the manufacturer’s protocol for TRIzol reagent. RNA samples were treated with DNase I (RQ1 DNase, Promega M6101) for 30 min to remove DNA contamination. RNA purity and concentration were assessed using a NanoDrop spectrophotometer (Thermo Fisher Scientific). Reverse transcription of 5 μg of total RNA to cDNA was performed using the High-Capacity cDNA Reverse Transcription Kit (Applied Biosystems, Thermo Fisher Scientific, catalogue # 4 368 814) in a total reaction volume of 20 μL. The cDNA was diluted to a final volume of 150 μL. Primers for calpain 1 (*Capn1*), calpain 2 (*Capn2*), Ribosomal Protein L27 (*Rpl27*) and β2 microglobulin (*β2M*) were designed using primer3web version 4.1.0 (primer://primer3.ut.ee) based on the sequences obtained from NCBI (Table 1). Prior to real-time RT-qPCR analysis, the efficiency of amplification for all primer pairs was evaluated to ensure proper use of the ΔΔCT method. RT-qPCR reactions were performed on QuantStudio7 Pro (Applied Biosystems) in a 10 μL final volume comprised of 1 μL of cDNA template, 5 μL of 2X SYBR Green master mix (ThermoFisher Scientific, catalogue # 4367659), 0.5 μL of 10 μM forward and reverse primer mix and sterile water. Melting curve analysis was conducted to confirm the specificity of the real-time RT-qPCR reaction, which was programmed to include a melting profile immediately following the thermal cycling protocol. Mean CT values were used for ΔΔCT calculations. Fold differences in gene expression were calculated using the ΔΔCT method [88]. To ensure robust and reliable normalisation, gene expression levels were normalized to two reference genes, *Rpl27* and *β2M*, rather than a single reference gene. Stability of *Rpl27* and *β2M* expression was confirmed by comparing raw Ct values between male MJD and WT mice, with no differences were detected, and Ct values remained consistent within groups. The results are presented as the fold change of target gene expression relative to a reference sample and normalized to the reference gene.

**Table 1:** Primer sequences used for real-time quantitative reverse transcriptase polymerase chain reaction (RT-qPCR).

**Primer ID**	**Sequence**	**Forward or Reverse**
*Rpl27* - Forward	TCATGCCCACAAGGTACTCTGT	Forward
Rpl27 - Reverse	CTGGCCTTGCGCTTCAAA	Reverse
β2M - Forward	CCCCACTGAGACTGATACATACG	Forward
β2M - Reverse	CGATCCCAGTAGACGGTCTTG	Reverse
*Capn1* - Forward	ACCACATTTTACGAGGGCAC	Forward
*Capn1* - Reverse	GGATCTTGAACTGGGGGTTT	Reverse
*Capn2* - Forward	CCCCAGTTCATTATTGGAGG	Forward
*Capn2* - Reverse	AAGCTCTGATCTGGAGGCAC	Reverse

### Statistical analysis

Data analysis was performed using GraphPad Prism (version 9). For Cohort 1 (phenotype characterisation), two-way repeated measures analysis of variance was used to analyse neurological scores across different genotypes and ages (factor 1: genotype, factor 2: age), followed by Tukey’s post-hoc comparisons. Similarly, comparisons of cleaved αII-spectrin concentrations in plasma samples from different ages were analysed using a two-way repeated measures ANOVA (factor 1: genotype, factor 2: age) if samples were analysed using the same ELISA plate. When time course samples were run on different ELISA plates, a Student t-test was used to compare genotypes at that specific age. Densiometric analysis of protein expression in cerebellum or brainstem tissue from MJD mice and wildtype littermate controls was compared using a Student’s t-test. Pearson correlation coefficients (two-tailed) were used to examine the relationship between levels of cleaved αII-spectrin (either from plasma or cerebellar protein lysates) and other parameters such as inherited CAG length or neurological score. For Cohort 2 (short-term treatment study), data was analysed using a Student’s t-test. Statistically significant differences are defined as *P* < 0.05.

## Supplementary Material

Supplementary_Figure_1_V3_ddaf196

Supplementary_Figure_2_V3_ddaf196

Supplementary_Figure_3_V3_ddaf196

Supplementary_Figure_4_ddaf196

Supplementary_Figure_5_ddaf196

Supplementary_Figure_6_V3_ddaf196

Supplementary_Figure_7_V3_ddaf196

Supplementary_Figures_ddaf196

## References

[1] Durr A . Autosomal dominant cerebellar ataxias: polyglutamine expansions and beyond. The Lancet Neurology 2010;9:885–894. 10.1016/S1474-4422(10)70183-6. 20723845

[2] Costa Mdo C, Paulson HL. Toward understanding Machado-Joseph disease. Prog Neurobiol 2012;97:239–257. 10.1016/j.pneurobio.2011.11.006. 22133674 PMC3306771

[3] Rüb U, Schöls L, Paulson H. et al. Clinical features, neurogenetics and neuropathology of the polyglutamine spinocerebellar ataxias type 1, 2, 3, 6 and 7. Prog Neurobiol 2013;104:38–66. 10.1016/j.pneurobio.2013.01.001. 23438480

[4] LaGrappe D, Massey L, Kruavit A. et al. Sleep disorders among aboriginal Australians with Machado-Joseph disease: quantitative results from a multiple methods study to assess the experience of people living with the disease and their caregivers. Neurobiology of Sleep and Circadian Rhythms 2022;12:100075. 10.1016/j.nbscr.2022.100075. 35516836 PMC9062757

[5] Pedroso JL, Braga-Neto P, Martinez AR. et al. Sleep disorders in Machado-Joseph disease. Curr Opin Psychiatry 2016;29:402–408. 10.1097/YCO.0000000000000287. 27584711

[6] Kawai Y, Takeda A, Abe Y. et al. Cognitive impairments in Machado-Joseph disease. Arch Neurol 2004;61:1757–1760. 10.1001/archneur.61.11.1757. 15534186

[7] Braga-Neto P, Pedroso JL, Alessi H. et al. Cerebellar cognitive affective syndrome in Machado Joseph disease: Core clinical features. Cerebellum 2012;11:549–556. 10.1007/s12311-011-0318-6. 21975858

[8] Takiyama Y, Nishizawa M, Tanaka H. et al. The gene for Machado–Joseph disease maps to human chromosome 14q. Nat Genet 1993;4:300–304. 10.1038/ng0793-300. 8358439

[9] Kawaguchi Y, Okamoto T, Taniwaki M. et al. CAG expansions in a novel gene for Machado-Joseph disease at chromosome 14q32.1. Nat Genet 1994;8:221–228. 10.1038/ng1194-221. 7874163

[10] Matsumura R, Takayanagi T, Murata K. et al. Relationship of (CAG)nC configuration to repeat instability of the Machado-Joseph disease gene. Hum Genet 1996;98:643–645. 10.1007/s004390050276. 8931692

[11] Abe Y, Tanaka F, Matsumoto M. et al. CAG repeat number correlates with the rate of brainstem and cerebellar atrophy in Machado-Joseph disease. Neurology 1998;51:882–884. 10.1212/WNL.51.3.882. 9748049

[12] Bettencourt C, Santos C, Montiel R. et al. The (CAG)n tract of Machado-Joseph disease gene (ATXN3): a comparison between DNA and mRNA in patients and controls. Eur J Hum Genet 2010;18:621–623. 10.1038/ejhg.2009.215. 19935829 PMC2987309

[13] Maciel P, Gaspar C, DeStefano AL. et al. Correlation between CAG repeat length and clinical features in Machado-Joseph disease. Am J Hum Genet 1995;57:54–61. 7611296 PMC1801255

[14] Scheel H, Tomiuk S, Hofmann K. Elucidation of ataxin-3 and ataxin-7 function by integrative bioinformatics. Hum Mol Genet 2003;12:2845–2852. 10.1093/hmg/ddg297. 12944423

[15] Yang H, Li J-J, Liu S. et al. Aggregation of polyglutamine-expanded ataxin-3 sequesters its specific interacting partners into inclusions: implication in a loss-of-function pathology. Sci Rep 2014;4:6410. 10.1038/srep06410. 25231079 PMC5377324

[16] Jana NR, Nukina N. Misfolding promotes the ubiquitination of polyglutamine-expanded ataxin-3, the defective gene product in SCA3/MJD. Neurotox Res 2004;6:523–533. 10.1007/BF03033448. 15639784

[17] Evers MM, Toonen LJA, van Roon-Mom WMC. Ataxin-3 protein and RNA toxicity in spinocerebellar ataxia type 3: current insights and emerging therapeutic strategies. Mol Neurobiol 2014;49:1513–1531. 10.1007/s12035-013-8596-2. 24293103 PMC4012159

[18] Seidel K, Siswanto S, Fredrich M. et al. On the distribution of intranuclear and cytoplasmic aggregates in the brainstem of patients with spinocerebellar ataxia type 2 and 3. Brain Pathol 2017;27:345–355. 10.1111/bpa.12412. 27377427 PMC8028910

[19] Scherzed W, Brunt ER, Heinsen H. et al. Pathoanatomy of cerebellar degeneration in spinocerebellar ataxia type 2 (SCA2) and type 3 (SCA3). Cerebellum 2012;11:749–760. 10.1007/s12311-011-0340-8. 22198871

[20] Rüb U, Gierga K, Brunt ER. et al. Spinocerebellar ataxias types 2 and 3: degeneration of the pre-cerebellar nuclei isolates the three phylogenetically defined regions of the cerebellum. J Neural Transm (Vienna) 2005;112:1523–1545. 10.1007/s00702-005-0287-3. 15785863

[21] Koeppen AH . The neuropathology of spinocerebellar ataxia type 3/Machado-Joseph disease. Adv Exp Med Biol 2018;1049:233–241. 10.1007/978-3-319-71779-1_11. 29427106

[22] López-Otín C, Bond JS. Proteases: multifunctional enzymes in life and disease. J Biol Chem 2008;283:30433–30437. 10.1074/jbc.R800035200. 18650443 PMC2576539

[23] Gafni J, Hermel E, Young JE. et al. Inhibition of calpain cleavage of huntingtin reduces toxicity: ACCUMULATION OF CALPAIN/CASPASE FRAGMENTS IN THE NUCLEUS^*^. J Biol Chem 2004;279:20211–20220. 10.1074/jbc.M401267200. 14981075

[24] Hübener J, Weber JJ, Richter C. et al. Calpain-mediated ataxin-3 cleavage in the molecular pathogenesis of spinocerebellar ataxia type 3 (SCA3). Hum Mol Genet 2012;22:508–518. 10.1093/hmg/dds449. 23100324

[25] Diepenbroek M, Casadei N, Esmer H. et al. Overexpression of the calpain-specific inhibitor calpastatin reduces human alpha-synuclein processing, aggregation and synaptic impairment in [A30P]αSyn transgenic mice. Hum Mol Genet 2014;23:3975–3989. 10.1093/hmg/ddu112. 24619358 PMC4110482

[26] Vosler PS, Brennan CS, Chen J. Calpain-mediated signaling mechanisms in neuronal injury and neurodegeneration. Mol Neurobiol 2008;38:78–100. 10.1007/s12035-008-8036-x. 18686046 PMC2726710

[27] Ono Y, Saido TC, Sorimachi H. Calpain research for drug discovery: challenges and potential. Nat Rev Drug Discov 2016;15:854–876. 10.1038/nrd.2016.212. 27833121

[28] Hanna RA, Campbell RL, Davies PL. Calcium-bound structure of calpain and its mechanism of inhibition by calpastatin. Nature 2008;456:409–412. 10.1038/nature07451. 19020623

[29] Baudry M, Bundman MC, Smith EK. et al. Micromolar calcium stimulates proteolysis and glutamate binding in rat brain synaptic membranes. Science 1981;212:937–938. 10.1126/science.7015504. 7015504

[30] Park S-Y, Ferreira A. The generation of a 17 kDa neurotoxic fragment: an alternative mechanism by which tau mediates β-amyloid-induced neurodegeneration. J Neurosci 2005;25:5365–5375. 10.1523/JNEUROSCI.1125-05.2005. 15930385 PMC1352316

[31] Russo R, Berliocchi L, Adornetto A. et al. Calpain-mediated cleavage of Beclin-1 and autophagy deregulation following retinal ischemic injury in vivo. Cell Death Dis 2011;2:e144–e144. 10.1038/cddis.2011.29. 21490676 PMC3122060

[32] De Marco G, Lomartire A, Manera U. et al. Effects of intracellular calcium accumulation on proteins encoded by the major genes underlying amyotrophic lateral sclerosis. Sci Rep 2022;12:395. 10.1038/s41598-021-04267-8. 35013445 PMC8748718

[33] Wang KKW . Calpain and caspase: can you tell the difference? Trends Neurosci 2000;23:20–26. 10.1016/S0166-2236(99)01479-4. 10652545

[34] Mishizen-Eberz AJ, Guttmann RP, Giasson BI. et al. Distinct cleavage patterns of normal and pathologic forms of α-synuclein by calpain I in vitro. J Neurochem 2003;86:836–847. 10.1046/j.1471-4159.2003.01878.x. 12887682

[35] Dufty BM, Warner LR, Hou ST. et al. Calpain-cleavage of α-synuclein: connecting proteolytic processing to disease-linked aggregation. Am J Pathol 2007;170:1725–1738. 10.2353/ajpath.2007.061232. 17456777 PMC1854966

[36] Saito K, Elce JS, Hamos JE. et al. Widespread activation of calcium-activated neutral proteinase (calpain) in the brain in Alzheimer disease: a potential molecular basis for neuronal degeneration. Proc Natl Acad Sci USA 1993;90:2628–2632. 10.1073/pnas.90.7.2628. 8464868 PMC46148

[37] Rao MV, Campbell J, Palaniappan A. et al. Calpastatin inhibits motor neuron death and increases survival of hSOD1G93A mice. J Neurochem 2016;137:253–265. 10.1111/jnc.13536. 26756888 PMC4828294

[38] Gafni J, Ellerby LM. Calpain activation in Huntington's disease. J Neurosci 2002;22:4842–4849. 10.1523/JNEUROSCI.22-12-04842.2002. 12077181 PMC6757710

[39] Weber JJ, Anger SC, Pereira Sena P. et al. Calpains as novel players in the molecular pathogenesis of spinocerebellar ataxia type 17. Cell Mol Life Sci 2022;79:262. 10.1007/s00018-022-04274-6. 35482253 PMC9050766

[40] Goti D, Katzen SM, Mez J. et al. A mutant Ataxin-3 putative-cleavage fragment in brains of Machado-Joseph disease patients and transgenic mice is cytotoxic above a critical concentration. J Neurosci 2004;24:10266–10279. 10.1523/JNEUROSCI.2734-04.2004. 15537899 PMC6730179

[41] Haacke A, Hartl FU, Breuer P. Calpain inhibition is sufficient to suppress aggregation of polyglutamine-expanded Ataxin-3^*^. J Biol Chem 2007;282:18851–18856. 10.1074/jbc.M611914200. 17488727

[42] Koch P, Breuer P, Peitz M. et al. Excitation-induced ataxin-3 aggregation in neurons from patients with Machado-Joseph disease. Nature 2011;480:543–546. 10.1038/nature10671. 22113611

[43] Watchon M, Yuan KC, Mackovski N. et al. Calpain inhibition is protective in Machado-Joseph disease zebrafish due to induction of autophagy. J Neurosci 2017;37:7782–7794. 10.1523/JNEUROSCI.1142-17.2017. 28687604 PMC6596655

[44] Weber JJ, Golla M, Guaitoli G. et al. A combinatorial approach to identify calpain cleavage sites in the Machado-Joseph disease protein ataxin-3. Brain 2017;140:1280–1299. 10.1093/brain/awx039. 28334907

[45] Simões AT, Gonçalves N, Koeppen A. et al. Calpastatin-mediated inhibition of calpains in the mouse brain prevents mutant ataxin 3 proteolysis, nuclear localization and aggregation, relieving Machado-Joseph disease. Brain 2012;135:2428–2439. 10.1093/brain/aws177. 22843411

[46] Gamage HKAH, Robinson KJ, Luu L. et al. Machado Joseph disease severity is linked with gut microbiota alterations in transgenic mice. Neurobiol Dis 2023;179:106051. 10.1016/j.nbd.2023.106051. 36822548

[47] Riederer BM, Lopresti LL, Krebs KE. et al. Brain spectrin(240235) and brain spectrin(240235E): conservation of structure and location within mammalian neural tissue. Brain Res Bull 1988;21:607–616. 10.1016/0361-9230(88)90200-6. 3208148

[48] Zhang Z, Larner SF, Liu MC. et al. Multiple alphaII-spectrin breakdown products distinguish calpain and caspase dominated necrotic and apoptotic cell death pathways. Apoptosis 2009;14:1289–1298. 10.1007/s10495-009-0405-z. 19771521

[49] Miller JA, Drouet DE, Yermakov LM. et al. Distinct changes in calpain and Calpastatin during PNS myelination and demyelination in rodent models. Int J Mol Sci 2022;23:15443. 10.3390/ijms232315443. PMC9737575 36499770

[50] Melloni E, De Tullio R, Averna M. et al. Properties of calpastatin forms in rat brain. FEBS Lett 1998;431:55–58. 10.1016/S0014-5793(98)00724-8. 9684864

[51] Ray SK, Neuberger TJ, Deadwyler G. et al. Calpain and calpastatin expression in primary oligodendrocyte culture: preferential localization of membrane calpain in cell processes. J Neurosci Res 2002;70:561–569. 10.1002/jnr.10414. 12404510

[52] Goll DE, Thompson VF, Li H. et al. The calpain system. Physiol Rev 2003;83:731–801. 10.1152/physrev.00029.2002. 12843408

[53] Yamashita T, Hideyama T, Hachiga K. et al. A role for calpain-dependent cleavage of TDP-43 in amyotrophic lateral sclerosis pathology. Nat Commun 2012;3:1307. 10.1038/ncomms2303. 23250437

[54] Tan CF, Yamada M, Toyoshima Y. et al. Selective occurrence of TDP-43-immunoreactive inclusions in the lower motor neurons in Machado-Joseph disease. Acta Neuropathol 2009;118:553–560. 10.1007/s00401-009-0552-x. 19526244

[55] Onofre I, Mendonça N, Lopes S. et al. Fibroblasts of Machado Joseph disease patients reveal autophagy impairment. Sci Rep 2016;6:28220. 10.1038/srep28220. 27328712 PMC4916410

[56] Sittler A, Muriel M-P, Marinello M. et al. Deregulation of autophagy in postmortem brains of Machado-Joseph disease patients. Neuropathology 2018;38:113–124. 10.1111/neup.12433. 29218765

[57] Watchon M, Luu L, Plenderleith SK. et al. Autophagy function and benefits of autophagy induction in models of spinocerebellar ataxia type 3. Cells 2023;12:893. 10.3390/cells12060893. 36980234 PMC10047838

[58] Yamashita T, Kwak S. The molecular link between inefficient GluA2 Q/R site-RNA editing and TDP-43 pathology in motor neurons of sporadic amyotrophic lateral sclerosis patients. Brain Res 2014;1584:28–38. 10.1016/j.brainres.2013.12.011. 24355598

[59] Robinson KJ, Tym MC, Hogan A. et al. Flow cytometry allows rapid detection of protein aggregates in cellular and zebrafish models of spinocerebellar ataxia 3. Dis Model Mech 2021;14. 10.1242/dmm.049023. PMC8524651 34473252

[60] Robinson KJ, Yuan K, Plenderleith SK. et al. A novel calpain inhibitor compound has protective effects on a zebrafish model of spinocerebellar ataxia type 3. Cells 2021;10. 10.3390/cells10102592. PMC8533844 34685571

[61] Watchon M, Wright AL, Ahel HI. et al. Spermidine treatment: induction of autophagy but also apoptosis? Molecular Brain 2024;17:15. 10.1186/s13041-024-01085-7. 38443995 PMC10916058

[62] Silva-Fernandes A, Duarte-Silva S, Neves-Carvalho A. et al. Chronic treatment with 17-DMAG improves balance and coordination in a new mouse model of Machado-Joseph disease. Neurotherapeutics 2014;11:433–449. 10.1007/s13311-013-0255-9. 24477711 PMC3996110

[63] Wellington CL, Hayden MR. Of molecular interactions, mice and mechanisms: new insights into Huntington's disease. Curr Opin Neurol 1997;10:291–298. 10.1097/00019052-199708000-00003. 9266152

[64] Matos CA, Almeida LP, Nobrega C. Proteolytic cleavage of polyglutamine disease-causing proteins: revisiting the toxic fragment hypothesis. Curr Pharm Des 2017;23:753–775. 10.2174/1381612822666161227121912. 28025946

[65] Van Den Bosch L, Van Damme P, Bogaert E. et al. The role of excitotoxicity in the pathogenesis of amyotrophic lateral sclerosis. Biochim Biophys Acta (BBA) - Mol Basis Dis 2006;1762:1068–1082. 10.1016/j.bbadis.2006.05.002. 16806844

[66] Wright AL, Vissel B. The essential role of AMPA receptor GluR2 subunit RNA editing in the normal and diseased brain. Front Mol Neurosci 2012;5. 10.3389/fnmol.2012.00034. PMC3324117 22514516

[67] Sobolevsky AI, Rosconi MP, Gouaux E. X-ray structure, symmetry and mechanism of an AMPA-subtype glutamate receptor. Nature 2009;462:745–756. 10.1038/nature08624. 19946266 PMC2861655

[68] Robinson KJ, Watchon M, Laird AS. Aberrant cerebellar circuitry in the spinocerebellar ataxias. Front Neurosci 2020;14:707. 10.3389/fnins.2020.00707. 32765211 PMC7378801

[69] Xia Z, Dudek H, Miranti CK. et al. Calcium influx via the NMDA receptor induces immediate early gene transcription by a MAP kinase/ERK-dependent mechanism. J Neurosci 1996;16:5425–5436. 10.1523/JNEUROSCI.16-17-05425.1996. 8757255 PMC6578897

[70] Berridge MJ, Irvine RF. Inositol trisphosphate, a novel second messenger in cellular signal transduction. Nature 1984;312:315–321. 10.1038/312315a0. 6095092

[71] Kasumu A, Bezprozvanny I. Deranged calcium Signaling in Purkinje cells and pathogenesis in spinocerebellar ataxia 2 (SCA2) and other ataxias. Cerebellum 2012;11:630–639. 10.1007/s12311-010-0182-9. 20480274 PMC3257360

[72] de la Fuente S, Sansa A, Hidalgo I. et al. Calpain system is altered in survival motor neuron-reduced cells from in vitro and in vivo spinal muscular atrophy models. Cell Death Dis 2020;11:487. 10.1038/s41419-020-2688-5. 32587237 PMC7316821

[73] Zhang YJ, Xu YF, Dickey CA. et al. Progranulin mediates caspase-dependent cleavage of TAR DNA binding protein-43. J Neurosci 2007;27:10530–10534. 10.1523/JNEUROSCI.3421-07.2007. 17898224 PMC6673167

[74] Wirawan E, Vande Walle L, Kersse K. et al. Caspase-mediated cleavage of Beclin-1 inactivates Beclin-1-induced autophagy and enhances apoptosis by promoting the release of proapoptotic factors from mitochondria. Cell Death Dis 2010;1:e18. 10.1038/cddis.2009.16. 21364619 PMC3032505

[75] Nelson WB, Smuder AJ, Hudson MB. et al. Cross-talk between the calpain and caspase-3 proteolytic systems in the diaphragm during prolonged mechanical ventilation. Crit Care Med 2012;40:1857–1863. 10.1097/CCM.0b013e318246bb5d. 22487998 PMC3358441

[76] Sharma AK, Rohrer B. Calcium-induced calpain mediates apoptosis via Caspase-3 in a mouse photoreceptor cell line^*^. J Biol Chem 2004;279:35564–35572. 10.1074/jbc.M401037200. 15208318

[77] Blomgren K, Zhu C, Wang X. et al. Synergistic activation of Caspase-3 by m-calpain after neonatal hypoxia-ischemia: a MECHANISM OF “PATHOLOGICAL APOPTOSIS”?^*^. J Biol Chem 2001;276:10191–10198. 10.1074/jbc.M007807200. 11124942

[78] Kupina NC, Detloff MR, Bobrowski WF. et al. Cytoskeletal protein degradation and neurodegeneration evolves differently in males and females following experimental head injury. Exp Neurol 2003;180:55–73. 10.1016/S0014-4886(02)00048-1. 12668149

[79] Silva-Fernandes A, Costa MC, Duarte-Silva S. et al. Motor uncoordination and neuropathology in a transgenic mouse model of Machado–Joseph disease lacking intranuclear inclusions and ataxin-3 cleavage products. Neurobiol Dis 2010;40:163–176. 10.1016/j.nbd.2010.05.021. 20510362

[80] Duarte-Silva S, Neves-Carvalho A, Soares-Cunha C. et al. Neuroprotective effects of creatine in the CMVMJD135 mouse model of spinocerebellar ataxia type 3. Mov Disord 2018;33:815–826. 10.1002/mds.27292. 29570846

[81] Esteves S, Duarte-Silva S, Naia L. et al. Limited effect of chronic valproic acid treatment in a mouse model of Machado-Joseph disease. PLoS One 2015;10:e0141610. 10.1371/journal.pone.0141610. 26505994 PMC4624233

[82] Esteves S, Oliveira S, Duarte-Silva S. et al. Preclinical evidence supporting early initiation of citalopram treatment in Machado-Joseph disease. Mol Neurobiol 2019;56:3626–3637. 10.1007/s12035-018-1332-1. 30173407

[83] Li M, Zhou S, Wang G. et al. Calpain inhibitor calpeptin improves Alzheimer’s disease–like cognitive impairments and pathologies in a diabetes mellitus rat model. Neurotox Res 2022;40:1248–1260. 10.1007/s12640-022-00561-z. 36018506

[84] Song Z-J, Yang S-J, Han L. et al. Postnatal calpeptin treatment causes hippocampal neurodevelopmental defects in neonatal rats. Neural Regen Res 2019;14:834–840. 10.4103/1673-5374.249231. 30688269 PMC6375038

[85] Li J, Yang S, Zhu G. Postnatal calpain inhibition elicits cerebellar cell death and motor dysfunction. Oncotarget 2017;8:87997–88007. 10.18632/oncotarget.21324. 29152136 PMC5675688

[86] Mayoral-Palarz K, Neves-Carvalho A, Duarte-Silva S. et al. Cerebellar neuronal dysfunction accompanies early motor symptoms in spinocerebellar ataxia type 3. Dis Model Mech 2022;15. 10.1242/dmm.049514. PMC9367011 35660856

[87] Li J, Grynspan F, Berman S. et al. Regional differences in gene expression for calcium activated neutral proteases (calpains) and their endogenous inhibitor calpastatin in mouse brain and spinal cord. J Neurobiol 1996;30:177–191. 10.1002/(SICI)1097-4695(199606)30:2<177::AID-NEU1>3.0.CO;2-2. 8738748

[88] Livak KJ, Schmittgen TD. Analysis of relative gene expression data using real-time quantitative PCR and the 2(-Delta Delta C(T)) method. Methods 2001;25:402–408. 10.1006/meth.2001.1262. 11846609

