# Supplementary figures and images for: Investigating the pathogenic role of calpain proteases and the therapeutic potential of their inhibition in mice modelling Machado-Joseph disease
Source: Hum Mol Genet. 2026 Jan 6;35(3):ddaf196. doi: 10.1093/hmg/ddaf196 (PMC13158246; doi:10.1093/hmg/ddaf196)

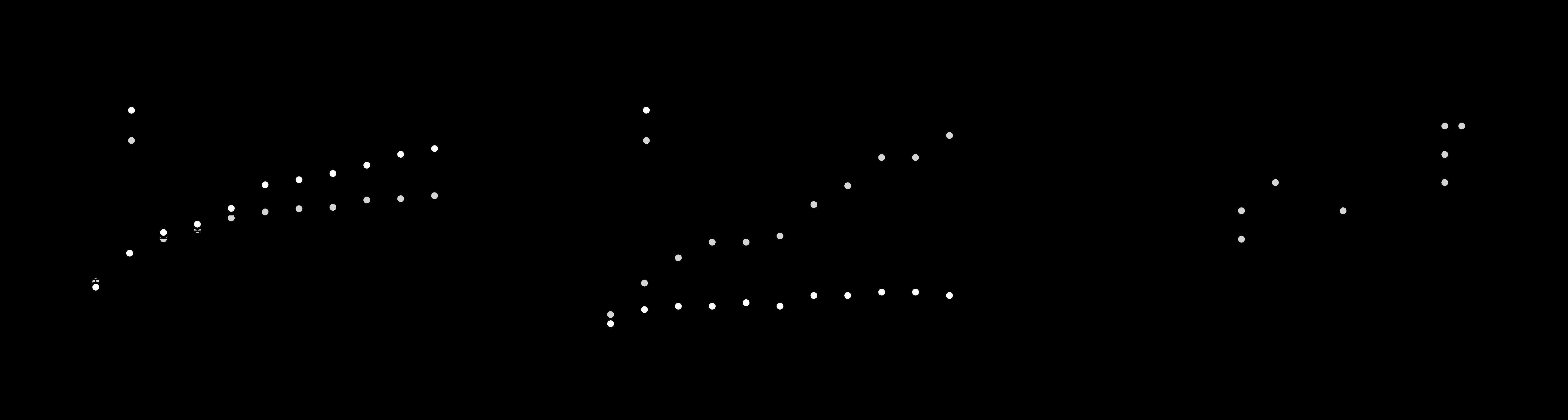

Supplement: Supplementary_Figure_1_V3_ddaf196 [file supplementary_figure_1_v3_ddaf196.jpeg]

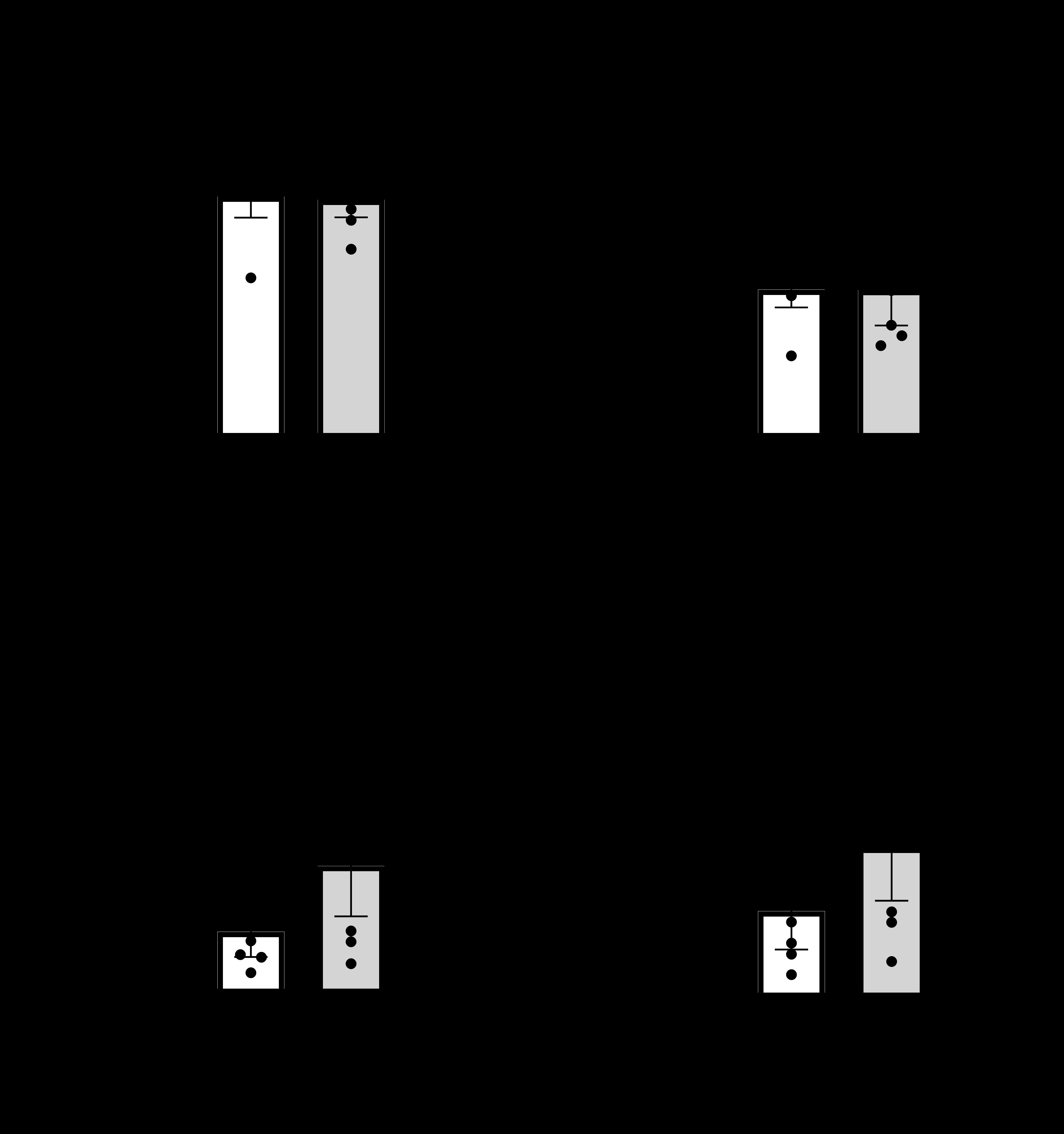

Supplement: Supplementary_Figure_2_V3_ddaf196 [file supplementary_figure_2_v3_ddaf196.jpeg]

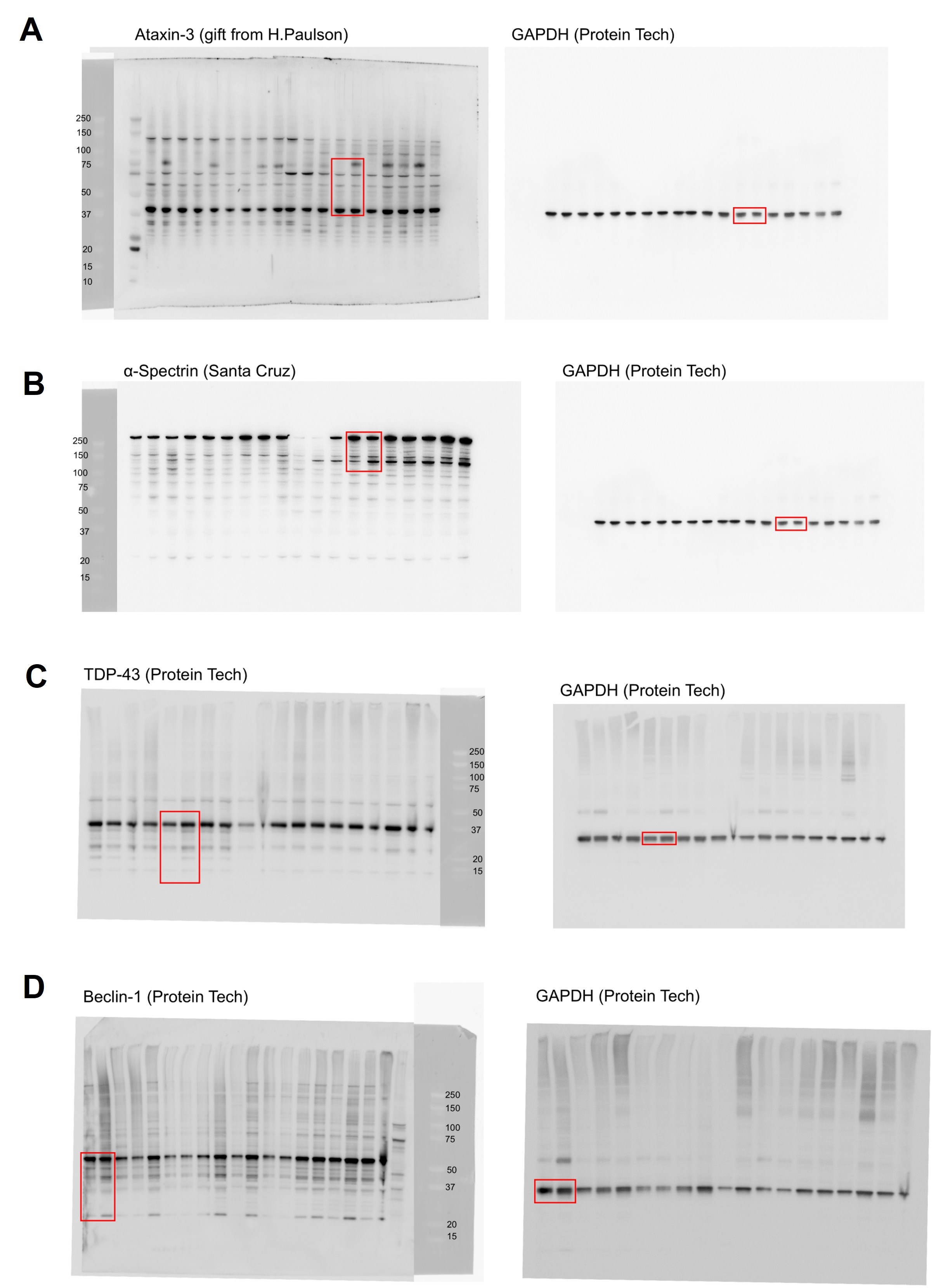

Supplement: Supplementary_Figure_3_V3_ddaf196 [file supplementary_figure_3_v3_ddaf196.jpeg]

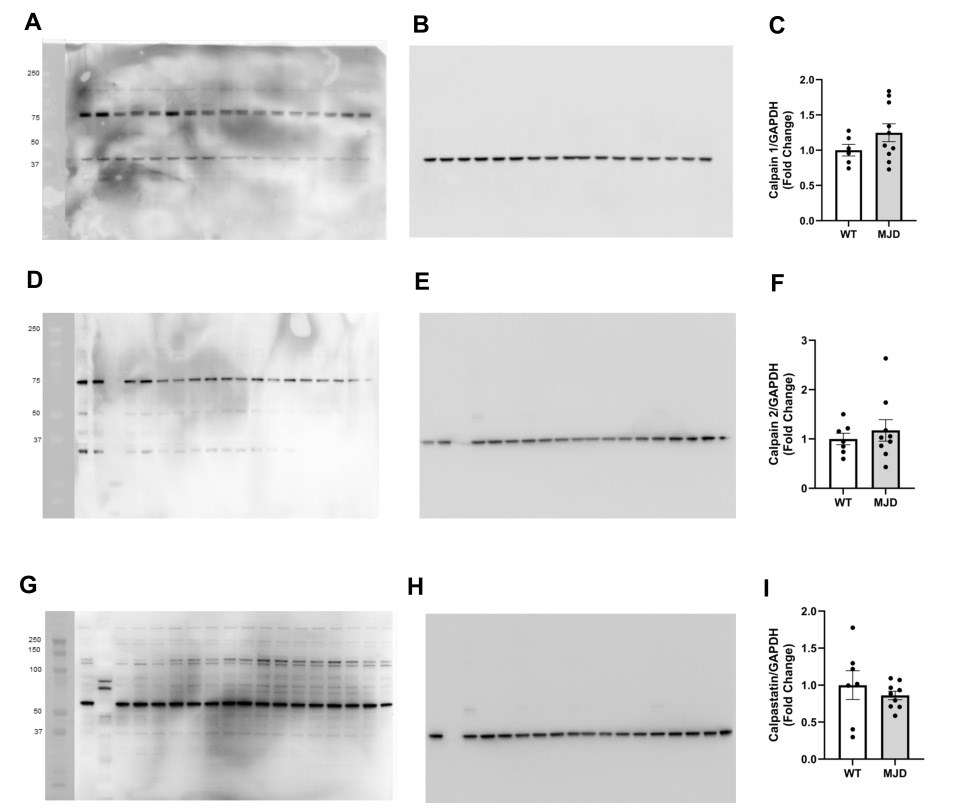

Supplement: Supplementary_Figure_4_ddaf196 [file supplementary_figure_4_ddaf196.jpeg]

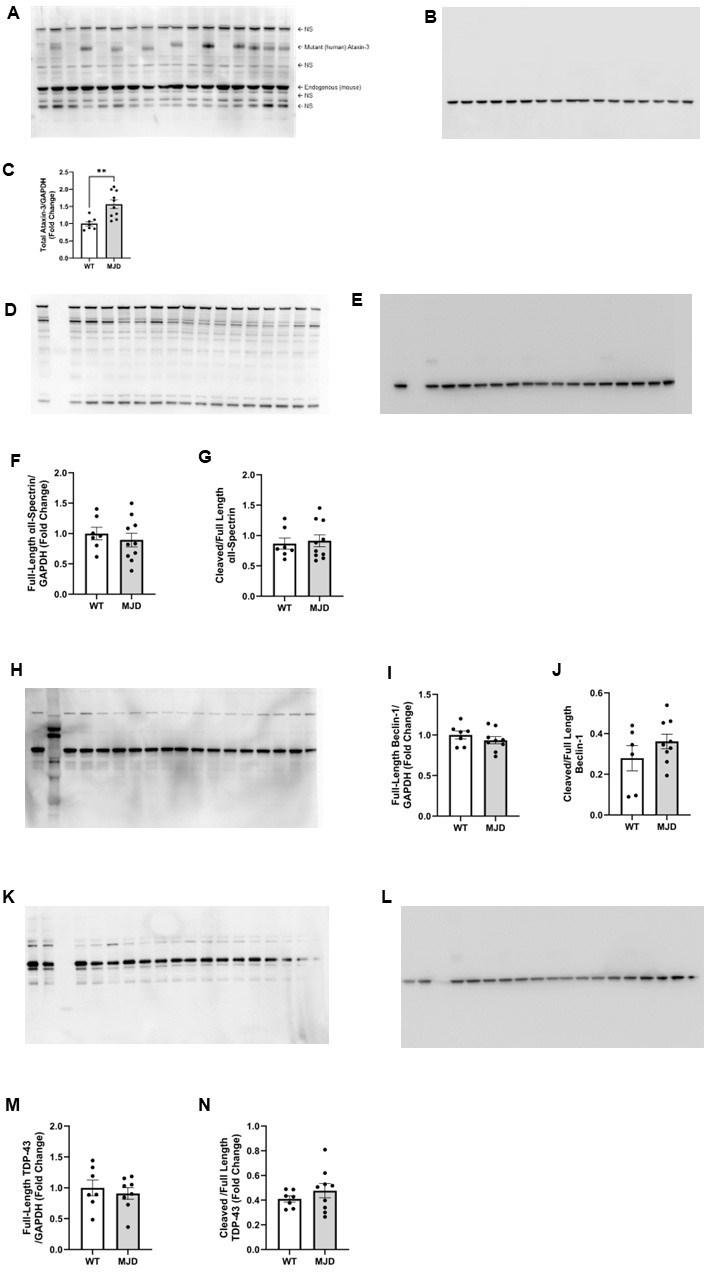

Supplement: Supplementary_Figure_5_ddaf196 [file supplementary_figure_5_ddaf196.jpeg]

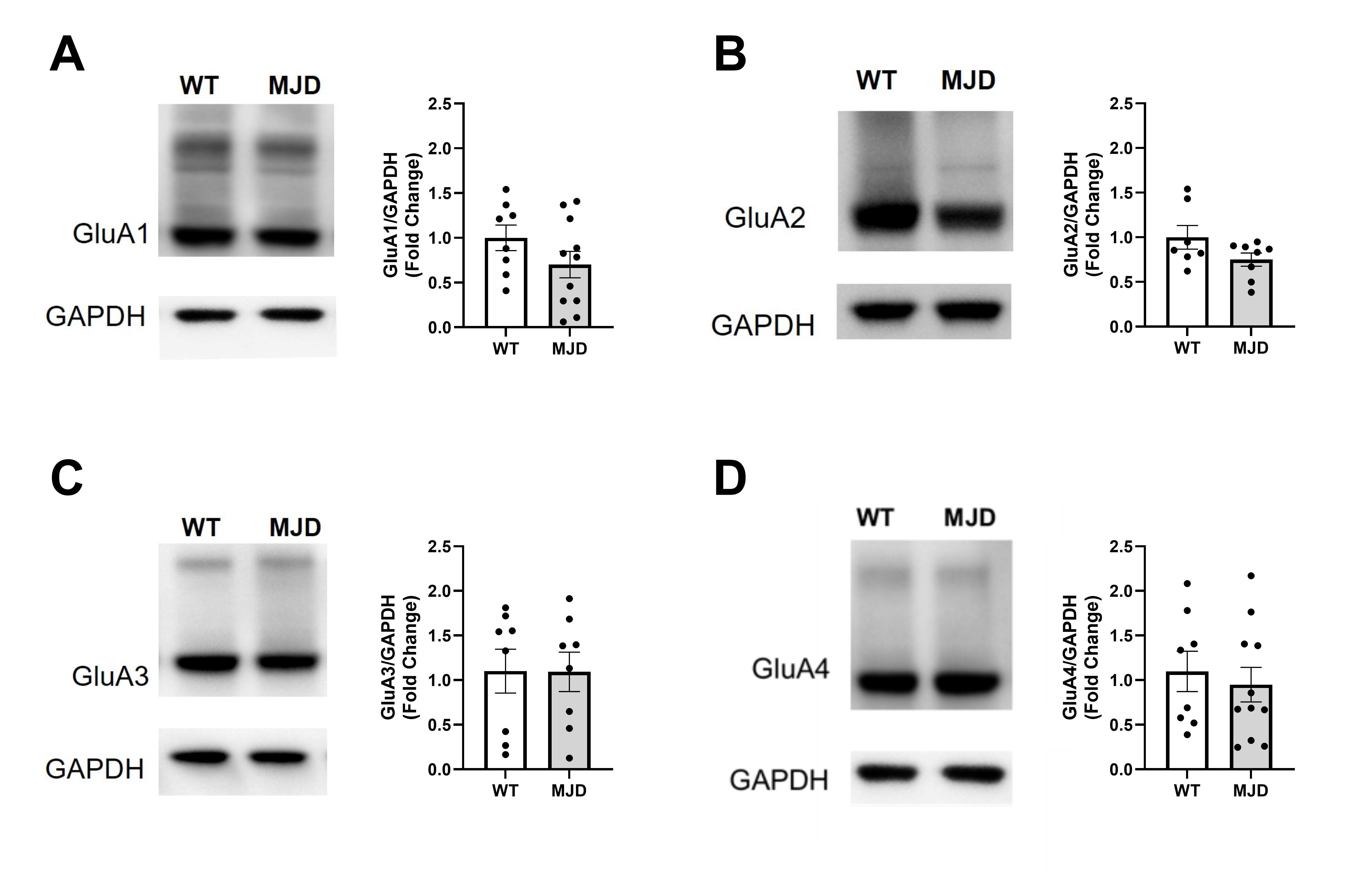

Supplement: Supplementary_Figure_6_V3_ddaf196 [file supplementary_figure_6_v3_ddaf196.jpeg]

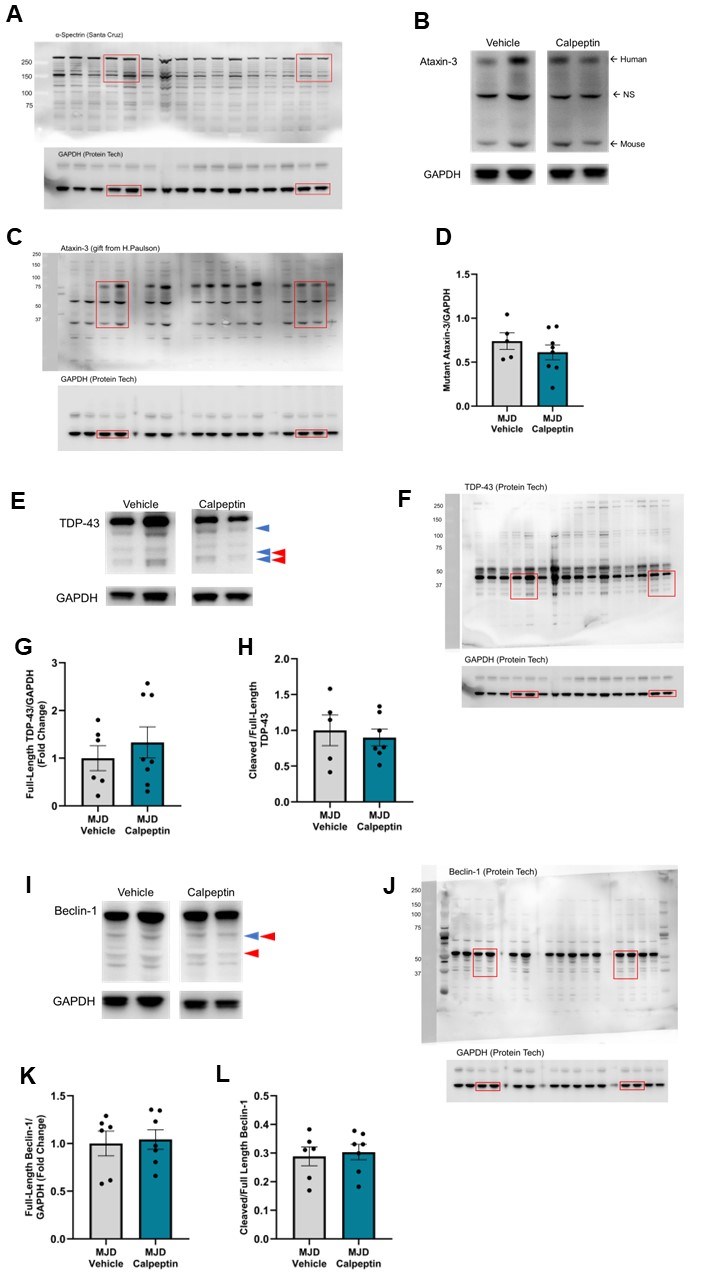

Supplement: Supplementary_Figure_7_V3_ddaf196 [file supplementary_figure_7_v3_ddaf196.jpeg]
