# Supplementary material for: Investigating the pathogenic role of calpain proteases and the therapeutic potential of their inhibition in mice modelling Machado-Joseph disease
Source: Hum Mol Genet. 2026 Jan 6;35(3):ddaf196. doi: 10.1093/hmg/ddaf196 (PMC13158246; doi:10.1093/hmg/ddaf196)
Supplement: Supplementary_Figures_ddaf196 [file supplementary_figures_ddaf196.docx]

**
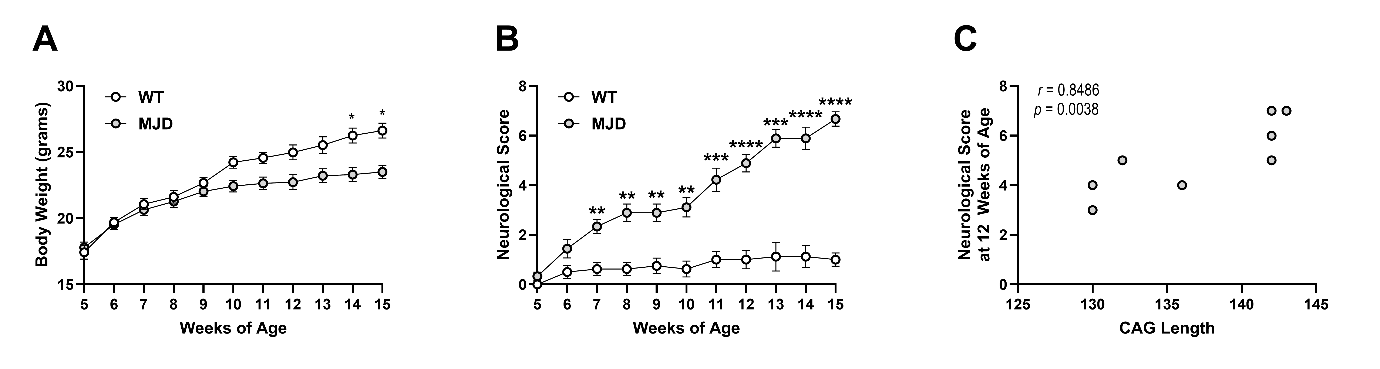
**

**Supplementary Figure 1.** Transgenic CMVMJD135 mice display slowed weight gain in comparison to wildtype littermates (A). CMVMJD135 develop neurological symptoms, as indicated by an increased neurological score, from 7 weeks of age (B). At 12 weeks of age, neurological score is positively correlated with inherited CAG repeat length in MJD mice (C). *n* = 9 MJD mice, *n* = 8 wildtype littermate controls. * represents *p* < 0.05, ** represents *p* < 0.01, *** represents *p* < 0.001 and **** represents *p* < 0.0001. Samples and data obtained from male mice only. Graphs are displaying the group mean ± SEM.


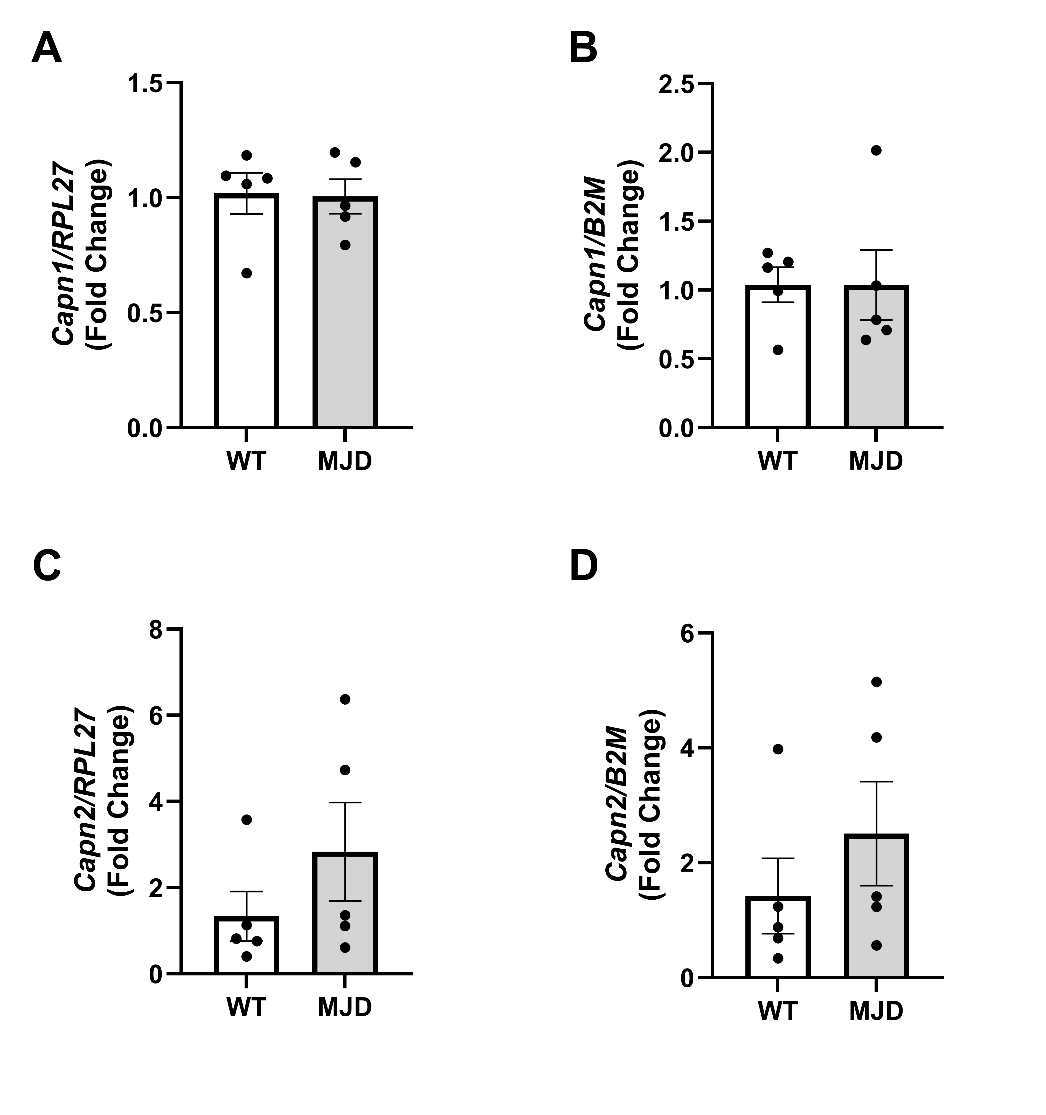


**Supplementary Figure 2.** Analysis of *Capn1* and *Capn2* gene expression in forebrain tissue. A-B) Levels of *Capn1* gene expression were not found to be altered across genotypes when normalising to reference genes *Rpl27* and *β2M*. C-D) Levels of Capn2 gene expression were not observed across genotypes when normalising expression to reference genes *Rpl27* or *β2M*. *n* = 5 animals per genotype. Samples and data obtained from male mice only. Graphs are displaying the group mean ± SEM.


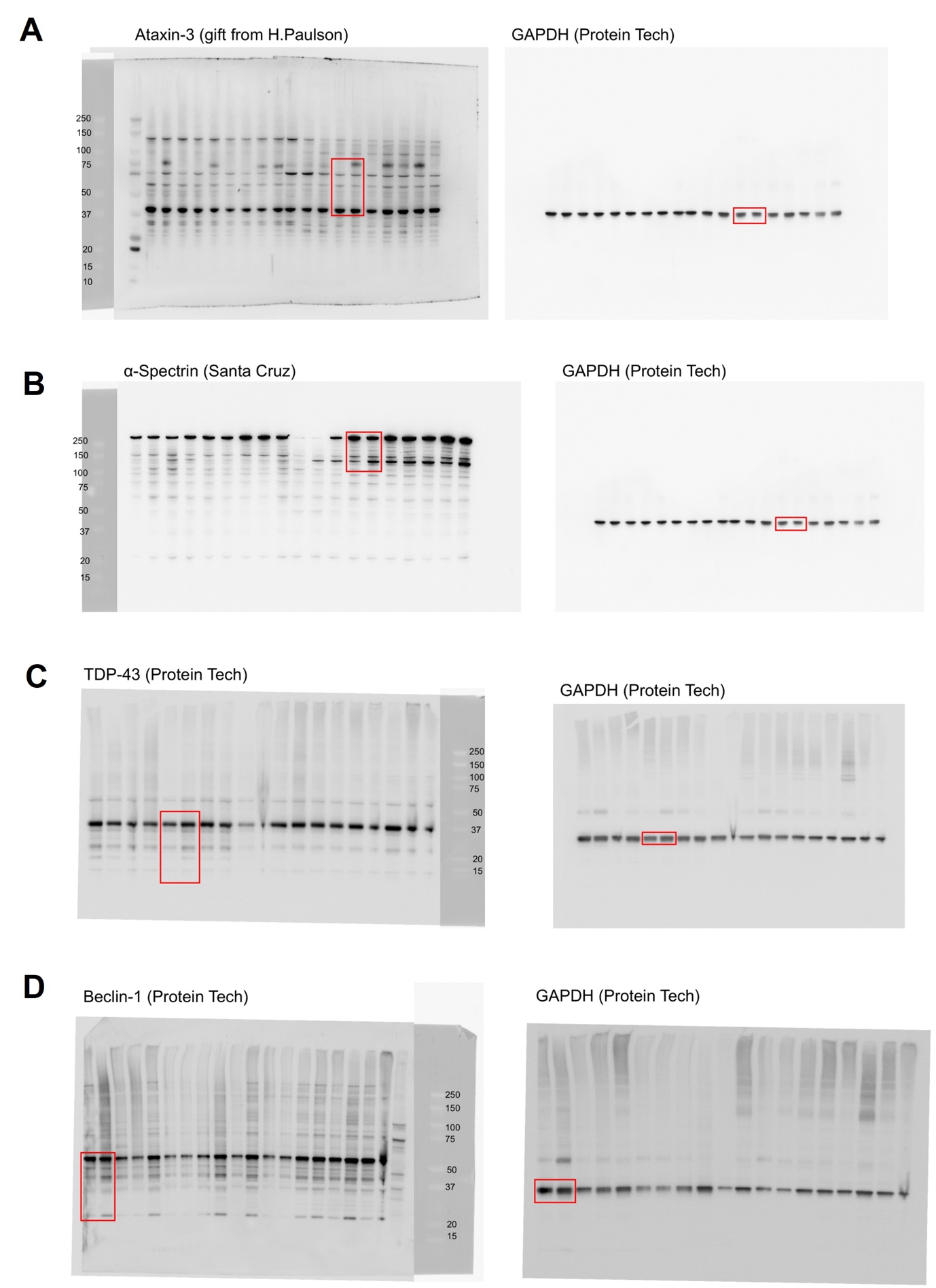


**Supplementary Figure 3.** Full western blot images of the representative westerns included in Figure 3. A) Full western blot image and molecular weight marker image used for the representative image depicted in Figure 3A. B) Full western blot image and molecular weight marker image used for representative image depicted in Figure 3C. C) Full western blot image and molecular weight marker image used for the representative image depicted in Figure 3I. D) Full western blot image and molecular weight marker image used for the representative image depicted in Figure 3L. Samples and data obtained from male mice only.


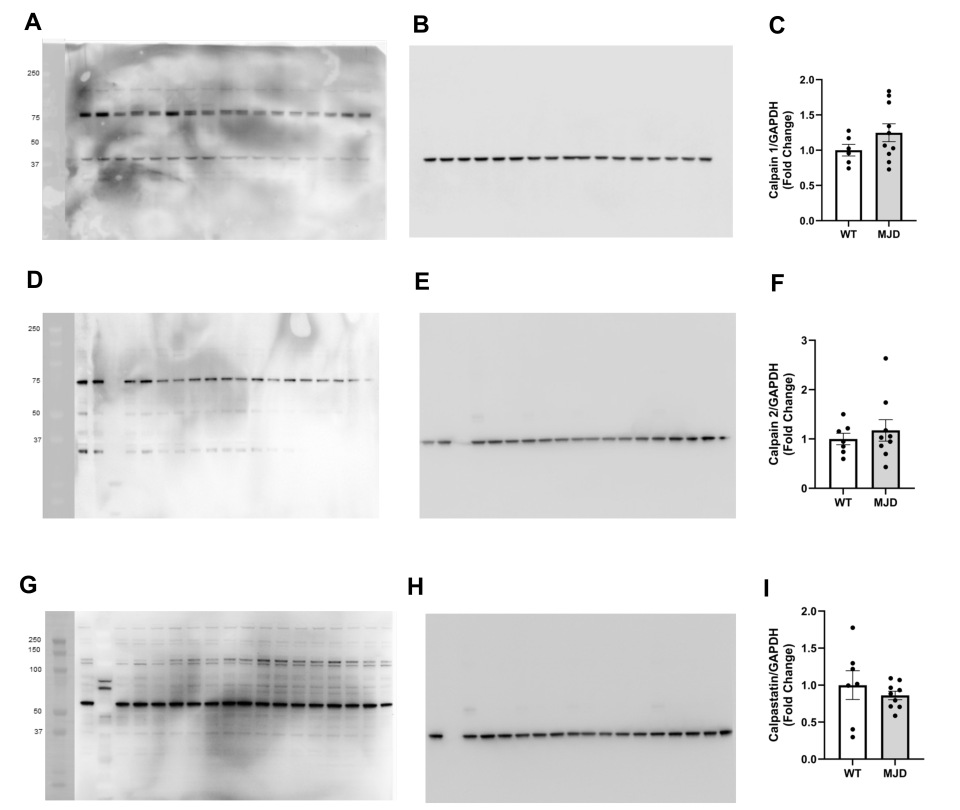


**Supplementary Figure 4.** Western blot analysis of calpain system proteins in brainstem lysates. A) Full western blot image of calpain 1 with molecular weight marker. B) Full western blot image of GAPDH immunoblot corresponding with the immunoblot image shown in A. C) Quantification of calpain 1 relative to GAPDH in brainstem protein lysates. D) Full western blot image of calpain 2 with molecular weight marker. E) Full western blot image of GAPDH immunoblot corresponding with immunoblot shown in D. F) Quantification of calpain 2 relative to GAPDH in brainstem protein lysates. G) Full western blot image of calpastatin and molecular weight marker. H) Full western blot image of GAPDH immunoblot corresponding to the image shown in G. I) Quantification of calpastatin relative to GAPDH in brainstem protein lysates. * represents *p* < 0.05. *n* = 6-10 per group. Samples and data obtained from male mice only. Graphs are displaying the group mean ± SEM.


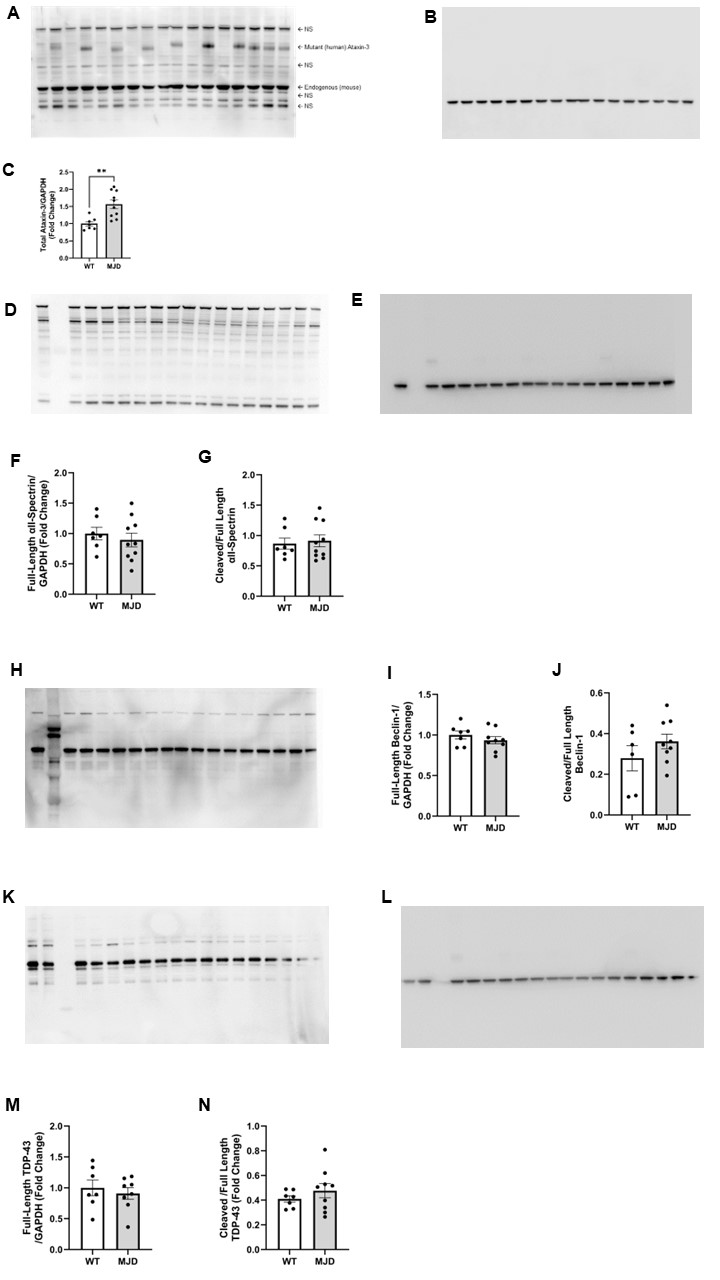


**Supplementary Figure 5.** Examination of calpain-mediated cleavage in brainstem protein lysates. A) Full western blot image of ataxin-3. B) Full western blot image of GAPDH immunoblot corresponding with the immunoblot shown in A. C) Quantification of total ataxin-3 relative to GAPDH in brainstem protein lysates. D) Full western blot image of αII-spectrin in brainstem lysates. E) Full western blot image of GAPDH immunoblot corresponding with the images shown in D and H. F) Quantification of full-length αII-spectrin relative to GAPDH in brainstem protein lysates. G) Quantification of cleaved αII-spectrin relative to full-length αII-spectrin in brainstem lysates. H) Full western blot image of beclin-1. I) Quantification of full-length beclin-1 relative to GAPDH. J) Quantification of cleaved beclin-1 relative to full-length beclin-1. K) Full western blot image of TDP-43. L) Full image of GAPDH levels in the same immunoblot shown in K. M) Quantification of full-length TDP-43 relative to GAPDH. N) Quantification of cleaved TDP-43 relative to full-length TDP-43. ** represents *p* < 0.01. *n* = 6-10 per group. Samples and data obtained from male mice only. Graphs are displaying the group mean ± SEM.


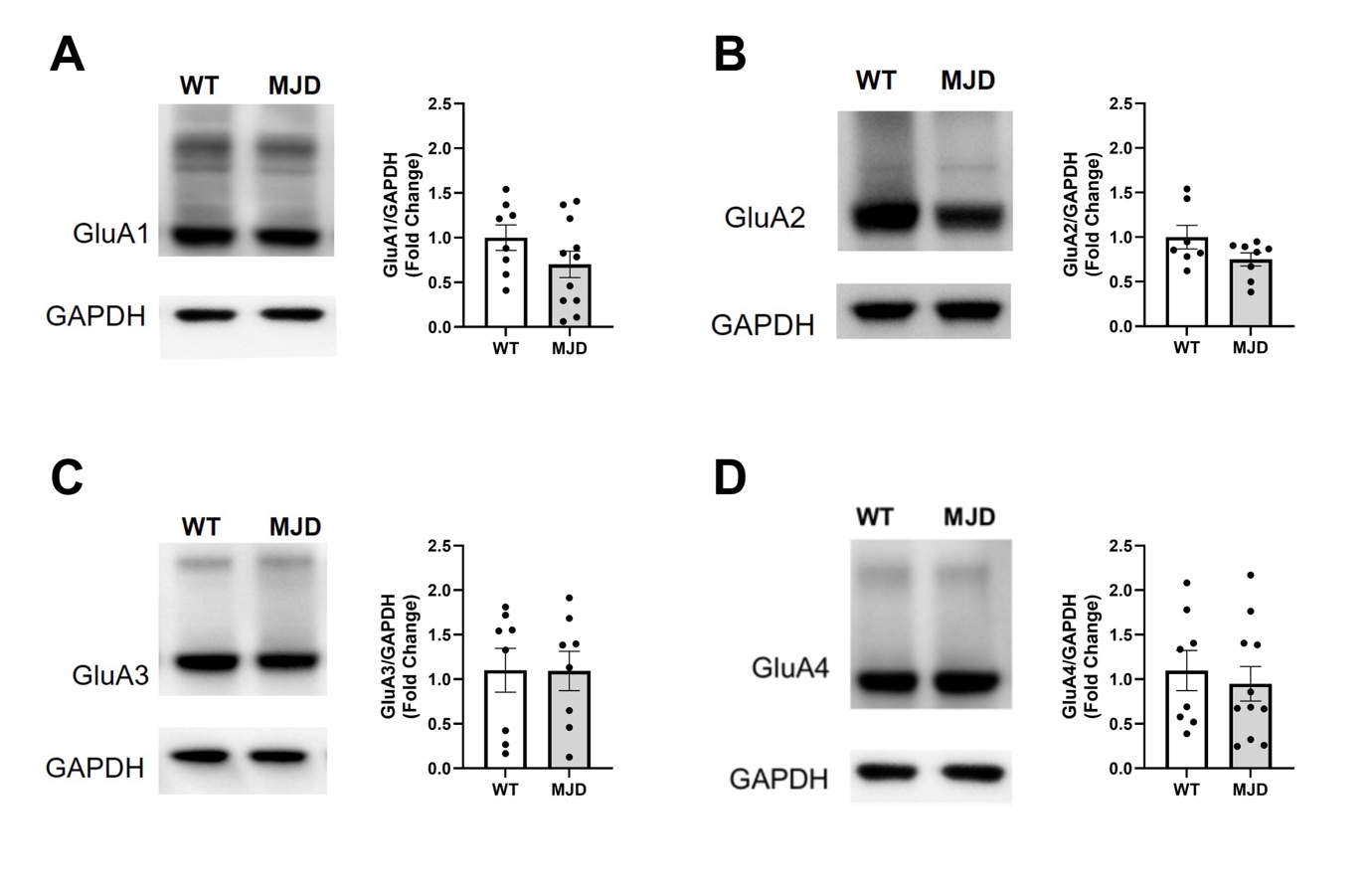


**Supplementary Figure 6.** Representative immunoblots and quantification of AMPA receptor subunit expression in cerebellum lysates from MJD mice and wildtype littermate controls. *n* = 8 wildtype mice, *n* = 7-11 MJD mice. Samples and data obtained from male mice only. Graphs are displaying the group mean ± SEM.


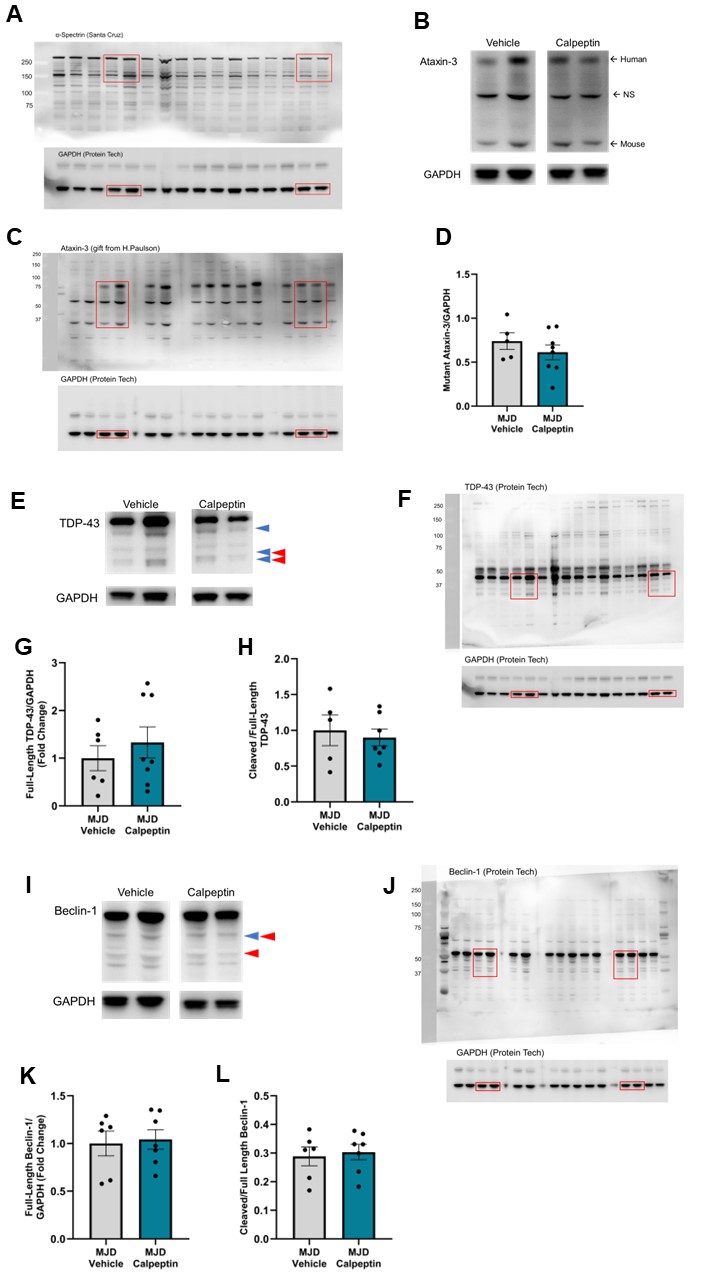


**Supplementary Figure 7**. Calpeptin treatment was not found to alter protein expression levels of cleavage of calpain substrates ataxin-3, TDP-43 or beclin-1. A) Full αII-spectrin western blot image, GAPDH image and molecular weight marker image depicted in Figure 6A. B) Representative western blot image displaying the presence of full-length mutant human ataxin-3 and endogenous mouse ataxin-3, with no cleavage fragments observed. C) Full ataxin-3 western blot image, GAPDH image and molecular weight marker image of ataxin-3. D) No statistically significant differences were detected when comparing mutant ataxin-3 protein levels across vehicle treated and calpeptin treated MJD animals. E) Representative immunoblot image displaying the presence of full-length TDP-43 and several TDP-43 cleavage fragments. F) Full TDP-43 western blot image, GAPDH image and molecular weight marker image. G) Densiometric analysis did not reveal any statistically significant difference in levels of full-length TDP-43 across treatment groups. H) The presence of TDP-43 cleavage fragments was found to be similar across vehicle treated and calpeptin treated MJD animals. I) Representative western blot image revealing the presence of full-length beclin-1 and beclin-1 cleavage fragments. J) Full beclin-1 western blot image, GAPDH image and molecular weight marker image. K) Protein levels of full-length beclin-1 relative to GAPDH were found to be similar across treatment groups. L) The ratio of cleaved beclin-1 relative to full-length beclin-1 was found to be similar across treatment groups. *n* = 5-6 vehicle treated MJD animals, *n* = 7-8 calpeptin treated MJD animals. Blue arrowheads represent cleavage fragments produced by calpain cleavage, red arrowheads represent cleavage fragments produced by caspase cleavage. Samples and data obtained from male mice only. Graphs are displaying the group mean ± SEM.
